# Shifts in surface microbiota after cleaning and disinfection in broiler processing plants: incomplete biofilm eradication revealed by robotic high-throughput screening

**DOI:** 10.1128/aem.02401-24

**Published:** 2025-02-26

**Authors:** Thorben Reiche, Gunhild Hageskal, Mihai Mares, Sunniva Hoel, Anne Tøndervik, Tonje Marita Bjerkan Heggeset, Tone Haugen, Sigri Bakken Sperstad, Hanne Hein Trøen, Solfrid Bjørkøy, Anita Nordeng Jakobsen

**Affiliations:** 1 Department of Biotechnology and Food Science, Norwegian University of Science and Technology 205785 , Trondheim, Norway; 2 Department of Biotechnology and Nanomedicine, SINTEF Industry 275243 , Trondheim, Norway; 3 Department of Public Health, "Ion Ionescu de la Brad" University of Life Sciences 162283 , Iași, Romania; 4 Norsk Kylling AS 669158 , Orkanger, Norway; Universita degli Studi di Napoli Federico II, Portici, Italy

**Keywords:** poultry, chicken, food processing, hygienic zone, *Pseudomonas*, metataxonomics, BacTiter-Glo, MIC

## Abstract

Broiler processing environments are a source of spoilage bacteria and potential pathogens. The aim of this study was to investigate the impact of cleaning and disinfection (C&D) on bacterial load and diversity in two broiler processing plants and to determine the efficacy of industry-standard disinfectants (DIs). C&D significantly reduced average bacterial loads on surfaces from 3.7 to 1.8 log CFU/cm^2^ in Plant A and from 7.0 to 3.8 log CFU/cm^2^ in Plant B (*P* < 0.001). Metataxonomics revealed that *Acinetobacter* and an unknown Enterobacteriaceae genus dominated before C&D in Plants A and B, respectively, while *Pseudomonas* was predominant after C&D in both plants. Bacterial diversity only declined significantly after C&D in Plant B. Bacterial loads also declined across hygienic zones along the broiler processing line in Plant A. During slaughter, *Staphylococcus*, *Lactobacillus*, and *Anoxybacillus* dominated, and bacterial loads were significantly higher compared with post-slaughter zones, which were dominated by *Pseudomonas*. Culture-dependent analyses confirmed the presence of *Pseudomonas* spp. and also bacteria affecting food safety, including *Listeria monocytogenes*, *Escherichia coli*, *Yersinia enterocolitica*, *Acinetobacter baumannii*, and *Pseudomonas aeruginosa*. A selection of these was tested against DIs using robotic high-throughput screening. At the recommended user concentrations, DIs effectively inhibited planktonic bacteria and significantly reduced mono-species biofilms. However, none of the DIs completely eradicated all biofilms at these concentrations, with survival rates ranging from 7% to 53%, depending on the DI. In conclusion, C&D effectively reduces the bacterial burden and reshapes the bacterial microbiota with incomplete biofilm eradication by commercial DIs.

**IMPORTANCE:**

Broiler meat continues to be involved in bacterial disease outbreaks. The surface microbiota in broiler processing environments can be a source of contaminating bacteria. Our study highlights the importance of effective C&D routines since potential pathogens and spoilage bacteria are found in these environments. Furthermore, the study provides evidence of biofilms surviving high concentrations of industry-standard DIs. This emphasizes the importance of additional measures to facilitate biofilm removal, such as mechanical cleaning, but also suggests that there is a need for DIs with stronger biofilm eradication capabilities. Ultimately, it is important to understand and continuously improve the state of hygiene in broiler processing plants to mitigate the risk of foodborne disease outbreaks.

## INTRODUCTION

Consumption of poultry meat is estimated to have the largest increase in the EU, while the consumption of beef and pork is expected to decline (1, 2). The annual consumption of poultry meat was 23.7 kg/capita in 2023, making it the second most consumed meat type. At the same time, poultry meat is commonly associated with foodborne diseases in the EU, with broiler meat identified as the leading food source of campylobacteriosis (3). With several outbreaks in 2022, e.g., in Denmark, France, and Spain, campylobacteriosis is one of the most common foodborne diseases in the EU. In Portugal, Romania, and Norway, no outbreaks were reported in the same year. However, the causative agent of campylobacteriosis, *Campylobacter,* is not the only pathogen that can be transmitted by broiler meat. In total, 21 strong-evidence foodborne disease outbreaks in the EU in 2022 were linked to broiler meat, of which *Listeria monocytogenes* was causative to most hospitalizations (97) and deaths (5) (3). Other pathogens that may be of risk for consumers include *Escherichia coli*, *Salmonella enterica*, *Staphylococcus aureus,* and *Enterococcus faecalis* (4–6). These may be transferred to the broiler meat during slaughter, processing, or packaging and can originate from breeding houses and the broiler’s intestinal tract and feathers (7, 8). Additionally, contaminating spoilage bacteria, such as lactic acid bacteria, *Pseudomonas*, and *Aeromonas*, may reduce the shelf-life of broiler products and contribute to food waste and economic losses (9). Controlling bacterial contaminants in primary production as well as mitigating cross-contamination during the slaughter and processing stages is therefore key to produce safe and high-quality broiler products.

The state of hygiene in broiler processing plants depends on many aspects, including the cleaning and disinfection (C&D) routines, hygienic design, maintenance of processing equipment, and staff hygiene (10). C&D reduces bacterial loads and controls the risks of bacterial contaminants. This is achieved by first removing any residual inorganic soils, i.e., dirt, and organic soils, such as carbohydrates, lipids, and proteins, as well as microorganisms (11). Thereafter, chemical disinfectants (DIs) are applied to destroy or reduce any residual microorganisms, which were not removed by cleaning. Successful removal can be challenging in the presence of biofilms, especially if these are located in areas difficult to access by C&D (12). Many studies have demonstrated that biofilms increase DI tolerance (13–16). Food safety issues can also occur since biofilms can aid the survival and persistence of pathogens (17, 18). The most frequently used DIs in the food industry include sodium hydroxide, quaternary ammonium compounds (QACs), sodium hypochlorite, peracetic acid, and hydrogen peroxide (19). The three latter DIs are causing oxidative damage to cellular components, while QACs are mainly causing protein denaturation and cell membrane disruption. Sodium hydroxide, in contrast, is a strong alkalic agent primarily causing damage by high pH. Exopolysaccharides in biofilms can, however, interfere with the active compounds of DIs and reduce their efficacy (20). As biofilms continue to be a challenge for the food industry, there is a need for more knowledge on the efficacy of commercial DIs to eradicate biofilms formed by bacteria relevant to specific processing environments.

Furthermore, dividing broiler processing plants into different hygienic zones can limit cross-contamination and bacterial transmission between different processing stages (21). These zones are designed to physically segregate the processing environment and strictly control the flow of staff, goods, tools, and processing equipment. Feces, intestines, and feathers can transfer large amounts of bacteria to the processing environment during slaughtering and may result in microbial hotspots in broiler processing plants (22). As a minimum, slaughtering should therefore be strictly segregated from the late processing stages, although further zoning may be advantageous. Different hygienic zones can be selective for certain types of bacteria, depending on the conditions in the processing environment (7). Investigating the microbiota in these zones can therefore help to understand bacterial challenges specific to the zone and to introduce measures if undesirable bacteria are frequently found. The industry also needs more knowledge on whether their C&D routines are effective in these zones and how they impact the surface microbiota. This knowledge is important to continuously improve the state of hygiene and ensure the production of safe and high-quality broiler products.

Here, we investigate the impact of C&D on bacterial loads and bacterial surface microbiota in broiler processing plants in Norway and Romania. Focus is on differences between hygienic zones, which include the transportation of live broiler, slaughtering, evisceration, processing, and packaging of broiler meat. The presence of foodborne pathogens and potential spoilage bacteria were analyzed, and the effect of commercial disinfectants were tested on a large selection of these bacteria. Bacterial isolates, including *Aeromonas veronii*, *Pseudomonas fluorescens*, *Pseudomonas aeruginosa*, *Escherichia coli*, and *Citrobacter freundii,* were tested both in planktonic and biofilm states using robotic high-throughput screening (HTS) technologies.

## MATERIALS AND METHODS

### Broiler processing plants

**Fig 1:**
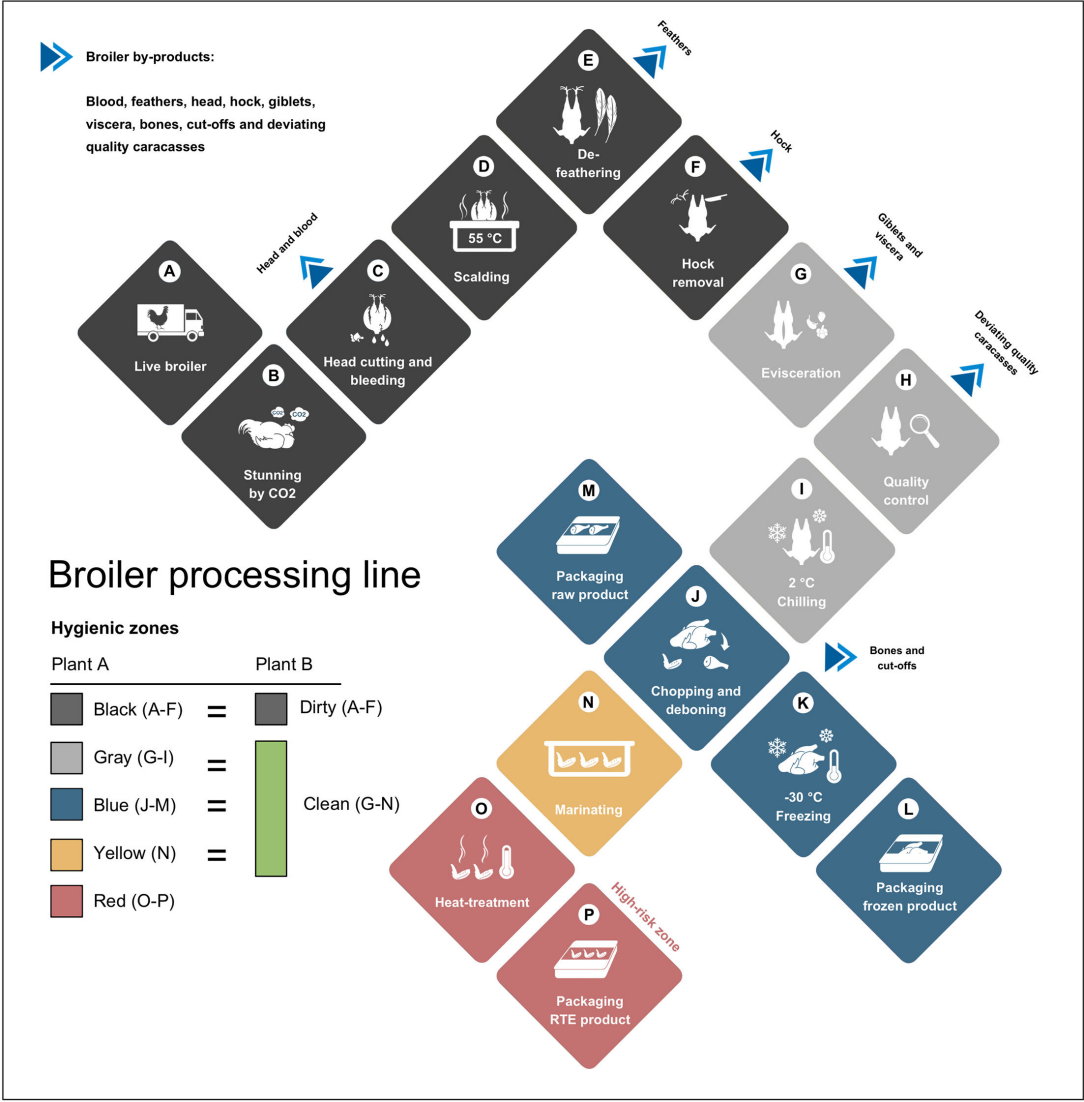
Overview of the broiler processing line (**A-P**) divided into different hygienic zones. The Norwegian broiler processing plant (Plant A) operates with five hygienic zones: black, gray, blue, yellow, and red, whereas the Romanian broiler processing plant (Plant B) is divided into a dirty and a clean zone. The dirty zone equals the black zone in Norway, i.e., the processing steps are similar. The clean zone in Romania equals the gray, blue, and yellow zone in Norway.

Two broiler processing plants were investigated as part of this study: one in Norway (Plant A) and one in Romania (Plant B). Both plants are highly automated with processing capacities of 130,000 and 100.000 broilers per day, respectively. The Norwegian broiler processing plant is divided into five different hygienic zones (Fig. 1). In the black zone (steps A–F), live broilers are received, stunned, head-cut, and scalded at 55°C for 133 s before feathers and hocks are removed. The processing continues in the gray zone (steps G–I) where the carcasses are eviscerated, quality checked, and chilled down to 2°C. Chopping, de-boning, weighing, and packaging take place in the blue zone (steps J–M), which produces raw and frozen broiler products. Some parts of the broiler are further processed in the yellow zone (step N) where brining and marinating take place. Finally, in the red zone (steps O–P), broiler products are heat treated, and ready-to-eat products are packaged (high-risk zone). The C&D routine starts with the application of a foaming detergent (3.5% Enduro Timesaver) with disinfecting properties and manual scrubbing. The foam is rinsed off with high-pressure hot water after a contact time of minimum 10 min. A sodium hypochlorite-based DI (3.5% Diverclean Hypofoam) is applied to food contact surfaces and drains with a contact time of minimum 15 min before a final rinsing step with water proceeds.

The Romanian broiler processing plant (Plant B) is divided into the dirty and clean hygienic zones. The dirty zone (steps A–F) equals the black hygienic zone from Plant A with a few differences, the scalding temperature is 59°C for 120 s, and head-cutting takes place after scalding. The clean zone (steps G–N) in Plant B equals the gray, blue, and yellow zone from Plant A, apart from the chilling temperature (step I), which is 8°C. Heat-treated products and ready-to-eat products are not produced in Plant B. The C&D routine in Plant B includes the following steps: pre-rinsing, application of detergent (3% Calgonit CT 312 or 3% Mida Foam 259 PN), rinsing, application of DI (3% Calgonit DS 680 or 0.25% Sanoxsept), rinsing, and drying. The contact time of detergents and DIs is 20 min. Concentrations are increased to 5% in especially dirty areas.

### Surface sampling before and after cleaning and disinfection

Cloth samples from 11 surfaces were collected once during the cold and warm seasons of 2022 (March and July) in the Norwegian broiler processing plant. In the Romanian broiler processing plant, 14 surfaces were cloth sampled twice during the cold season of 2022 (February and October) and once during the warm season of 2023 (May). The surfaces included both food contact surfaces (FCSs) and non-food contact surfaces (NFCSs), which were sampled during production before C&D and after C&D ahead of production the following day (Table 1). Details about the sampling method are described in Reiche et al. (23). In brief, approximately 30 × 30 cm was sampled using pre-moistened sterile cloths (Sodibox, France), which were homogenized in peptone water, centrifuged to collect pellets, and stored at −80°C until DNA extraction. Aliquots from the same homogenates were also used for selecting bacterial isolates and quantifying total aerobic bacteria, as described in the next section.

**TABLE 1:** Overview of surface sampling points in the production environment of a broiler processing plant in Norway (*n* = 11) and Romania (*n* = 14)^*a*^

Processing plant	Surface sampling point	Processing step	Contact surface	Hygienic zone	Explanation
Plant A Norway	Transport truck	A	NFCS	Black	Processing of live broiler, stunning, slaughter, scalding, and feather removal
	Transport boxes	A	NFCS	Black	
	Plucking machine	E	NFCS	Black	
	Organ extraction	G	FCS	Gray	Post-scalding processing, evisceration, and quality control
	Fat suction unit	G	FCS	Gray	
	Conveyor for trimming	J	FCS	Blue	Processing of broiler, deboning, sizing, and trimming
	Conveyor for multi-head weigher	J	FCS	Blue	
	Conveyor for trimming table	J	FCS	Blue	
	Deskinner	J	FCS	Blue	
	Inside brining vessel	N	FCS	Yellow	Marinating and brining of broiler meat
	Conveyor to filling machine	P	FCS	Red	Heat-treatment and packaging of RTE products
					
Plant B Romania	Defeathering area	E	NFCS	Dirty	Arrival of live broiler, stunning, slaughter, scalding, and feather removal
	Floors in defeathering area	E	NFCS	Dirty	
	Evisceration machine	G	FCS	Clean	Evisceration, deboning, sizing, trimming, and packaging of products
	Chilling transfer belt	I	FCS	Clean	
	Chilling transfer belt 2	I	FCS	Clean	
	Wings cutting module	J	FCS	Clean	
	Thigh deboning area	J	FCS	Clean	
	Breast calibration belt	J	FCS	Clean	
	Breast with bone machine	J	FCS	Clean	
	Floors in cutting area	J	NFCS	Clean	
	Breast slide machine	J	FCS	Clean	
	Vertical packaging machine	M	FCS	Clean	
	Packing hall belt	M	FCS	Clean	
	Packing hall belt 2	M	FCS	Clean	

^*a*^ Sampling points are divided by processing step (Fig. 1), contact surface, i.e., food contact surface (FCS) or non-food contact surface (NFCS), hygienic zone, and the explanation of these zones.

### Total aerobic bacteria and bacterial isolates

Total aerobic mesophilic bacteria were quantified by preparing 10-fold serial dilutions of homogenates from cloth surface samples and plating on plate count agar (PCA) (Oxoid, UK/Scharlab, Spain) with incubation conditions at 37 °C for 24 h. Quantification was performed during two sampling campaigns in Plant A and during one of the three samplings in Plant B. Boxplots for total aerobic bacteria were generated by R package ggplot2 (version 3.4.3) (24) with statistical comparisons by ggpubr (25) and multiple comparisons by Tukey post-hoc test (SPSS Statistics) (version 29.0.2).

Enterobacterales, *Pseudomonas*, and *Enterococcus* isolates were selected by plating homogenates from cloth surface samples from Plant A on Violet Red Bile Glucose Agar (VRBGA, Oxoid), *Pseudomonas* CFC selective agar with supplement (CFC, Oxoid), and Slanetz and Bartley Agar (S-B, Oxoid). The CFC plates were incubated at 25°C for 48 h., the VRBGA plates at 37°C for 24 h, and the S-B plates at 42°C for 24 to 48 h. Homogenates from Plant B were plated on RAPID'E.coli 2 (RPE), RAPID'P.aeruginosa (RPP), and RAPID'Enterococcus (RPC) provided by Bio-Rad, France. The RPE and RPP plates were incubated at 36°C for 24 h, while RPC plates were incubated at 44°C for 24 to 48 h.

Up to four colonies were picked from each plate and re-streaked twice or more on tryptic soy agar (TSA) or brain heart infusion agar (BHI) (Becton Dickinson, Germany/VWR, Belgium). Glycerol stocks were prepared with 20% glycerol and tryptic soy broth (TSB) or brain heart infusion broth (BHB) (Becton Dickinson/VWR) and stored at -80°C. Homogenates were also enriched by *Listeria* enrichment broth (LEB) with LEB-selective supplement (Oxoid) and incubated overnight. Samples from Plant A were confirmed by real-time PCR using the SureTect *Listeria* species PCR assay and *Listeria monocytogenes* PCR assay (Thermo Scientific, Finland). Samples from Plant B were confirmed by plating the overnight enrichment culture on RAPID'*L. mono* (Bio-Rad).

Bacterial isolates were identified by polymerase chain reaction (PCR) and Sanger sequencing as previously described in Reiche et al. (23), with PCR conditions also found in Table A4. In brief, DNA was extracted from bacterial isolates from Plant A (*n* = 266) and Plant B (*n* = 207) using the DNeasy Blood and Tissue kit (Qiagen, Germany). PCR was performed targeting the *rpoD* gene in presumptive *Pseudomonas* spp. and the 16S rRNA gene (V3-V9) in presumptive Enterobacterales, *Enterococcus*, and *Listeria* spp. Identified *Aeromonas* spp. by 16S rRNA were also subjected to PCR targeting *gyrB*. Sanger sequencing of PCR products was outsourced to Eurofins Genomics (Köln, Germany), and *in-silico* sequences were analyzed in the GenBank database using BLASTn (NCBI).

A phylogenetic tree was constructed for *Pseudomonas rpoD* sequences and relevant reference strains belonging to different groups and subgroups of the *Pseudomonas* genus (Table A5). The MUSCLE algorithm (26) was used for multiple sequence alignments in the MEGA11 software (version 11.0.13) (27) before constructing a phylogenetic tree using the neighbor-joining method (28). Evolutionary distances were calculated by the Kimura two-parameter method (29) with 1,000 bootstrap replicates, and visualization was performed by iTol (version 6.8) (30).

### Metataxonomics

Total DNA was extracted from pellets of cloth surface samples taken in Plant A using the MasterPure Gram Positive DNA Purification Kit (Biosearch Technologies, Novato, USA). Samples from Plant B were subjected to the OmniPrep kit for Gram-positive bacteria (G-Biosciences, St. Louis, USA). DNA extraction was performed according to the manufacturer’s protocols. DNA quantification, PCR, preparation of sequencing libraries, and sequencing conditions were performed as described previously by Reiche et al. (23), targeting the hypervariable V3–V4 region of 16S rRNA. Details on the bioinformatic analyses are also found in our previous publication. In brief, the CLC Genomics Workbench 24.0 (Qiagen, Denmark) was used for read trimming, filtering, and operational taxonomic unit (OTU) clustering using the Greengenes database with 97% similarity for classification and normalization for 16S rRNA copy numbers using the PICRUSt2 Multiplication table (31). Relative abundance plots and heatmaps were generated by R packages ampvis2 (version 2.8.3) (32), phyloseq (version 1.42.0) (33), ggplot2, and fantaxtic (version 0.2.1) (34). Both alpha- and beta-diversity indexes were calculated from the unnormalized OTU data using phyloseq and ggplot2. Significant differences (α = 0.05) were calculated by the Wilcoxon signed-rank test using ggpubr and adonis by vegan (version 2.6–6.1) (35) and pairwiseAdonis (version 0.4.1) (36).

### Efficacy of commercial disinfectants by robotic high-throughput assays

Minimum inhibitory concentration (MIC) testing and biofilm screening with commercial DIs were performed by robotic HTS protocols as previously described by Reiche et al. (23). For the MIC tests, *Pseudomonas* and Enterobacterales isolates from Plant A (n = 175) and Plant B (n = 138) were tested against three concentrations of eight commercial DIs (Table 2) (excluding Sanoxsept). In brief, three concentrations of each DI were prepared in Mueller Hinton II broth (cation adjusted) and arranged in 384-well plates in triplicates. DI plates were inoculated by diluted overnight cultures and incubated at 25 °C for 48 h (CFC isolates) or 37 °C for 24 h (remaining isolates). The MIC was defined as the lowest concentration that inhibits ≥70% of growth (OD_600_) as compared with the growth control.

For the biofilm screening, BacTiter GLO (BTG) assays were used to indirectly determine levels of adenosine triphosphate in biofilm growth controls and parallel biofilms exposed to three concentrations of eight DIs (Table 2) (excluding Ecas4). Triplicates of 62 mono-species biofilms were cultivated overnight using 96-well plates at 25 °C (CFC isolates) or 37 °C (remaining isolates). Supernatants were removed, and biofilms were washed twice by the robotic workstation (Biomek NXp) aspirating and dispensing liquids with precise control without dislodging biofilms on well bottoms. A small adjustment was made during washing procedures, i.e., waste reservoirs were replaced by 96-well plates for waste collection to minimize cross-contamination between tips. Biofilms were exposed to the DIs for 20 min and washed twice after exposure before adding the BTG reagent and measuring luminescence (RLU). If mean RLU values in exposed biofilms were 10 times greater and significantly higher than mean RLU in the background noise, it was considered as biofilm survival (Student’s t test, two-tailed, α = 0.01). Boxplots were generated by ggplot2 and ggpubr with multiple comparisons by Tukey post-hoc test (SPSS Statistics).

**TABLE 2:** Overview of commercial disinfectants included in this study

Disinfectant	Main active substances	Assay conc. (%)	Recommended user conc. by the producer (%)	Supplier
Aqua DES Foam PAA	Peracetic acid Hydrogen peroxide Acetic acid	0.5, 1, 2	1–2	Aquatic Chemistry AS, Lillehammer, Norway
Delladet VS2	Alkyl dimethylbenzyl ammonium chloride Alkyl alcohol ethoxylate	0.9, 1.8, 3.6	1–2	Lilleborg AS, Oslo, Norway
Enduro Timesaver	Potassium hydroxide, isotridecanol ethoxylate	2, 4, 8	2–8	Lilleborg AS, Oslo, Norway
Titan Hypo	Sodium hypochlorite Sodium hydroxide	1, 5, 7.5	0.5–1	Lilleborg AS, Oslo, Norway
Aqua Biocip	Potassium hydroxide Sodium hydroxide Sodium hypochlorite	0.3, 2, 5	0.3–2	Aquatic Chemistry AS, Lillehammer, Norway
Calgonit DS 680	Didecyldimethylammonium chloride Glutaraldehyde	0.015, 1, 3	1.5–3	Calvatis GmbH, Ladenburg, Germany
Ecas4^*a*^	Hypochlorous acid Redox potential: ~850 mV	4, 50, 100	100	Ecas4 Australia Pty. Ltd., Adelaide, Australia
Aqua San AM Plus	Alkylamine (N-(3-aminopropyl)-N-dodecylpropane-1)	1, 3, 5	0.5–2	Aquatic Chemistry AS, Lillehammer, Norway
Sanoxsept	Peracetic acid Hydrogen peroxide Acetic acid	0.15, 0.25, 0.50	0.15–0.25	SaneChem sp.z o.o. Poland

^*a*^ Ecas4 was produced in-house by a salmon processing company using an Ecas4 apparatus based on a membrane electrolytic cell.

## RESULTS

### Significant reductions in bacterial loads by cleaning and disinfection in broiler processing plants

**Fig 2:**
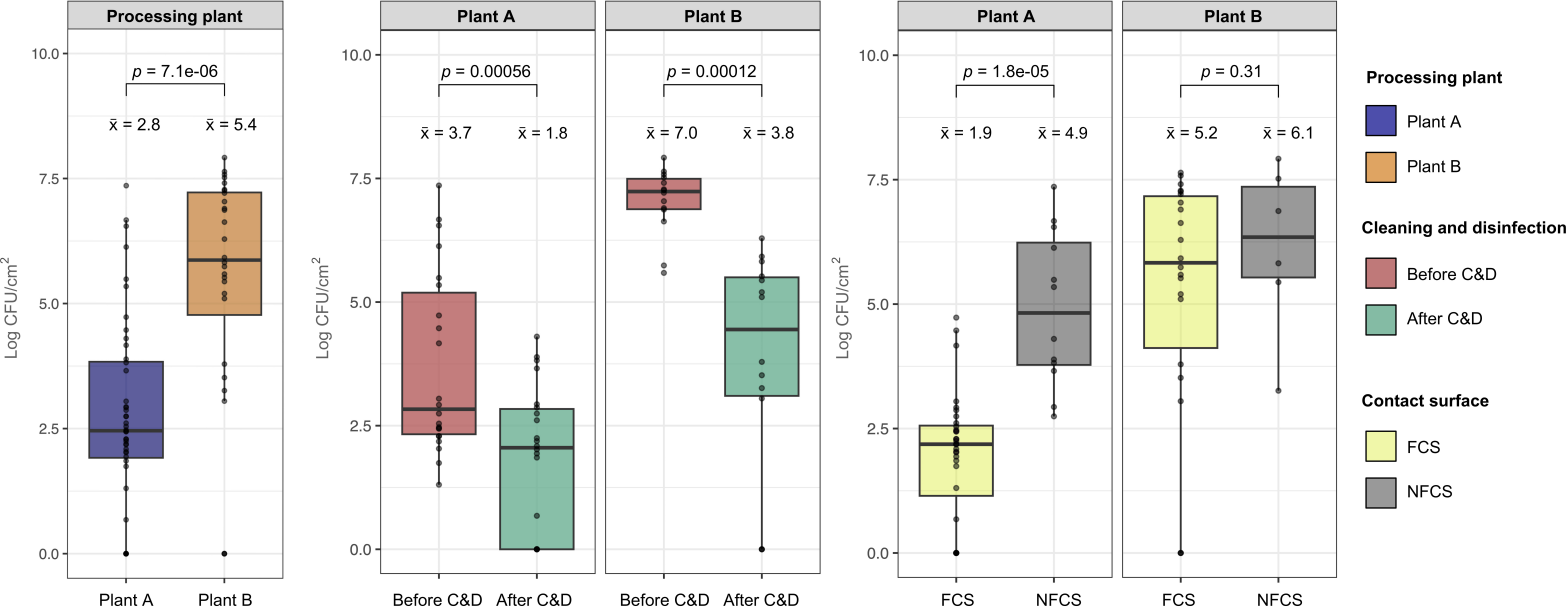
Boxplots showing total aerobic bacteria (log CFU/cm^2^) in samples from Plant A (*n* = 44) and Plant B (*n* = 28) comparing samples taken before or after C&D and on food contact surface (FCS) or non-food contact surfaces (NFCS). The median is indicated by a horizontal line in each box, and mean values are shown by x̄, while *P* values for significant differences are given by the Student’s *t*-test (two-tailed).

To investigate the effect of C&D on bacterial loads in two broiler processing plants, we collected surface samples from both food contact surfaces (FCSs) and non-food contact surfaces (NFCSs). We found significant reductions (*P* < 0.001) in bacterial loads by C&D in both plants (Fig. 2). The average total aerobic bacteria on surfaces in Plant A were 3.7 log CFU/cm^2^ before C&D and 1.8 log CFU/cm^2^ after C&D, resulting in an average log reduction of 1.9 log units. The overall average bacterial counts were significantly higher in Plant B, ranging from 7.0 log CFU/cm^2^ before C&D to 3.8 log CFU/cm^2^ after C&D. The average log reduction was also larger in Plant B compared with Plant A, equivalent to more than 3 log units. In both processing plants, we also found a few surfaces where no aerobic bacteria were detected after C&D. These include the breast slide machine in Plant B and the conveyor to the filling machine and the brining vessels in Plant A. Overall, these results suggest that the C&D routines are effective in reducing the bacterial loads in both processing plants.

**Fig 3:**
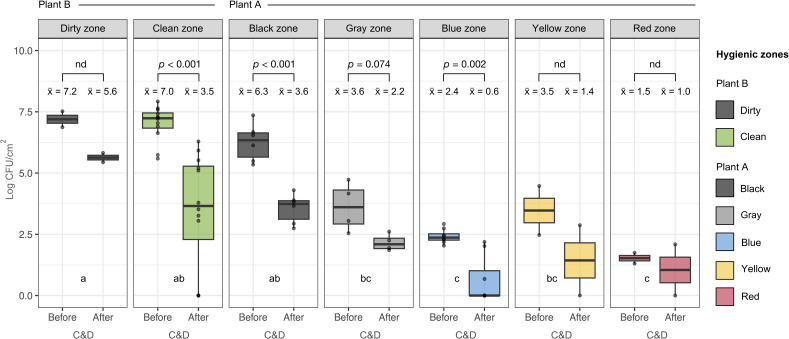
Boxplots showing total aerobic bacteria (log CFU/cm^2^) in samples taken before and after C&D in different hygienic zones. These include the dirty and clean zones in plant B and the black, gray, blue, yellow, and red zones in Plant A. The median is indicated by a horizontal line in each box, and mean values are shown by x̄, while *P* values for significant differences are given by the Student’s *t*-test (two-tailed). Different letters (**A, B**) show significant differences (*P* = 0.05) between hygienic zones, including samples taken both before and after C&D (calculated by Tukey post-hoc test).

Furthermore, we investigated differences between NFCSs, such as transport trucks, defeathering areas and floors, and FCSs, which include processing machines, conveyors for broiler meat and brining vessels (Fig. 2) On average, the bacterial loads were higher on NFCSs compared with FCSs in both processing plants ranging from 4.9 to 1.9 log CFU/cm^2^ in Plant A and 6.1 to 5.2 log CFU/cm^2^ in Plant B, respectively, although the difference was only significant in Plant A. Likewise, we only observed significant differences in Plant A when comparing bacterial loads in different hygienic zones (Fig. 3). Both the blue and red zones had significantly lower bacterial counts compared with the black, gray, and yellow zones (Tukey post-hoc test, *P* > 0.05). The lowest counts were found in the red zone, where ready-to-eat products are handled (high-risk zone). Counts were also low in the yellow zone after C&D, but relatively high before C&D and, therefore, overall, not significantly different from the black and gray zone. C&D also reduced bacterial loads all the other hygienic zones, with significant differences in the black and blue zones from Plant A and in the clean zone from Plant B. Significant differences could not be calculated for the red, yellow, and dirty zones because they were only sampled twice. Overall, we observed a clear decline in the bacterial loads from the start to the end of the broiler processing line in Plant A, suggesting that bacterial carryover from one hygienic zone to another is minimized. This is especially important when bacterial loads are high during early processing steps.

### Classification of bacterial isolates from broiler processing plants revealed a diverse consortium of bacterial taxa

Bacterial taxa affecting the spoilage and safety of broiler products were isolated by culture dependent methods from surfaces in the two broiler processing plants. During the two sampling campaigns in Plant A, we detected presumptive *Pseudomonas* spp. on 86% and 84% of surfaces sampled before and after C&D, respectively (Fig. A1 and A2). Presumptive Enterobacterales and *Enterococcus* spp. were detected on 64% and 68% of surfaces sampled before C&D and 18% and 41% of surfaces sampled after C&D, respectively. Most of the Enterobacterales and *Enterococcus* spp. were detected in the black and gray hygienic zones, i.e., transport trucks and areas where broilers are slaughtered. *L. monocytogenes* was only detected in the brining vessel before C&D, contrary to Plant B, where *L. monocytogenes* was not detected on any surface (Fig. A2). Presumptive *Pseudomonas* spp. were detected on 49% and 13% of surfaces sampled before and after C&D, respectively, whereas presumptive Enterobacterales and *Enterococcus* spp. were detected on 79% and 62% of surfaces sampled before C&D and 31% and 26% of surfaces sampled after C&D, respectively. The results from Plant B are, however, not directly comparable to results from Plant A since different selective media were used.

Furthermore, we identified 103 *Pseudomonas* isolates, 71 Enterobacterales isolates, 69 *Enterococcus* isolates, and 23 other isolates among the 266 isolates collected in Plant A (Tables A1 to A3), while 42 *Pseudomonas* isolates, 96 Enterobacterales isolates, 44 *Enterococcus* isolates and 25 other isolates were identified among the 207 isolates sampled in Plant B. Among the *Pseudomonas* isolates from Plants A and B, 91 and 33 isolates were successfully classified by *rpoD*, and the remaining isolates were classified by 16S rRNA sequencing. In Plant A, the most frequently detected species were *P. fluorescens* (24%), *P. lundensis* (12%), *P. putida* (9%), *P. fragi* (7%), and *P. coleopterorum* (4%) based on taxonomic classification of *rpoD* sequences using BLASTn (NCBI Genbank). The most frequently detected species in Plant B were *P. aeruginosa* (30%), *P. koreensis* (24%), *P. mosselii* (12%), *P. fluorescens* (12%), and *P. monteilii* (6%).

**Fig 4:**
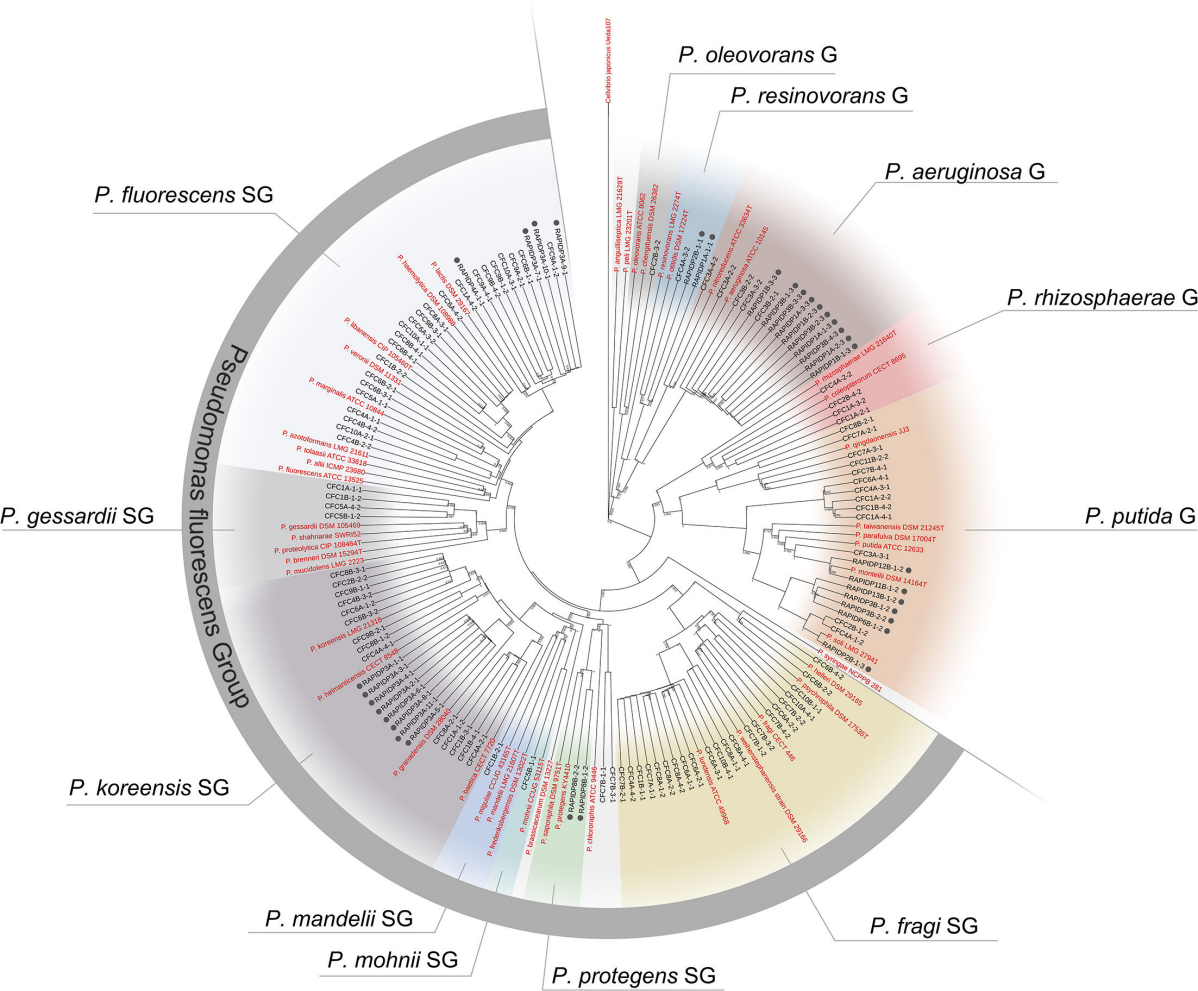
Neighbor-joining tree based on *rpoD*-sequences of *Pseudomonas* spp. isolated from two broiler processing plants. Isolates from Plant B (*n* = 33) are marked by black dots, while the remaining isolates are from Plant A (*n* = 91). Taxa are divided into groups (G) and subgroups (SG) based on 49 reference strains (marked in red) belonging to these groups and obtained from the GenBank database. A more detailed version of the tree is found in Fig. A3.

Classification by phylogenetic analyses revealed that 74% of the *Pseudomonas* isolates from Plant A belonged to the *P. fluorescens* group (Fig. 4). The remaining isolates were found in the groups of *P. putida* (14%), *P. aeruginosa* (5%), *P. rhizosphaerae* (4%), *P. resinovorans* (1%), and *P. oleovorans* (1%). Isolates from the latter four groups were only found in the black and gray hygienic zones, whereas isolates in the *P. putida* and *P. fluorescens* groups were found in almost all hygienic zones in Plant A. Within the *P. fluorescens* group, the isolates were further classified into subgroups of *P. fluorescens* (34%), *P. fragi* (33%), *P. koreensis* (21%), and *P. gessardii* (6%). *Pseudomonas* isolates collected from Plant B were also found in several of the groups and subgroups in the phylogenetic tree, including the *P. fluorescens* (42%), *P. putida* (21%), *P. aeruginosa* (30%), and *P. resinovorans* groups (6%). Similar to Plant A, *P. aeruginosa* was only detected during the early processing steps, i.e., the dirty zone. A connection between which species was found before or after C&D was not identified.

The most frequently detected species among the 71 Enterobacterales isolates from Plant A were *E. coli* (42%), *Serratia liquefaciens* (10%), *Hafnia paralvei* (7%), and *Klebsiella michiganensis* (7%). Only one *E. coli* isolate was found after C&D, and only two were found outside of the gray and black hygienic zones. Most of the *Hafnia* isolates were found in the blue zone, while *S. liquefaciens* were the only Enterobacterales isolates found in the red zone (high-risk zone). Isolates found in the yellow zone, the brining vessel, include *K. michiganensis*, *Proteus vulgaris*, and *Raoultella ornithinolytica*. The top identified species among the 96 Enterobacterales isolates from Plant B were *E. coli* (41%), *C. freundii* (14%), *Enterobacter kobei* (5%), and *Citrobacter braakii* (5%). Opposing to Plant A, *E. coli* was found during both early and late processing steps in Plant B on 10 out of 14 surfaces sampled before C&D. *E. coli* was not detected on any of these surfaces after C&D. Potential pathogens, such *Yersinia enterocolitica* and *Shigella dysenteriae* were also detected in Plant B. However, 16S rRNA species-level taxonomy should be interpreted with caution.

The majority of the 69 *Enterococcus* isolates from Plant A belonged to three species, *E. faecalis* (54%), *E. faecium* (32%), and *E. hirae* (7%). *E. faecium* was only found in the black and blue zones, while *E. faecalis* was found in all hygienic zones except the yellow zone. Mostly detected before C&D, but several isolates were also found after C&D, including one isolate sampled on the conveyor to the filling machine in the red zone where ready-to-eat products are packaged (high-risk zone). Among the 44 *Enterococcus* isolates from Plant B, *E. faecalis* was also the most identified (93%) and detected at most of the surfaces sampled. Only one *E. faecium* isolate was found. Most of the remaining 23 other isolates from Plant A belong to three *Aeromonas* species: *A. caviae* (22%), *A. jandaei* (4%), and *A. veronii* (4%). These were only found in the black zone both before and after C&D. Similarly, most of the remaining 25 other isolates from Plant B also belong to *Aeromonas* species, *A. veronii* (40%), *A. media* (24%), *A. hydrophila* (8%), *A. salmonicida* (4%), and *A. allosaccharophila* (4%), found both in the dirty and clean zones before and after C&D. Overall, the classification of bacterial isolates from the broiler processing plants revealed a diverse consortium of bacterial taxa.

### Cleaning and disinfection significantly affected relative abundances in the bacterial surface microbiota found in broiler processing plants

**Fig 5:**
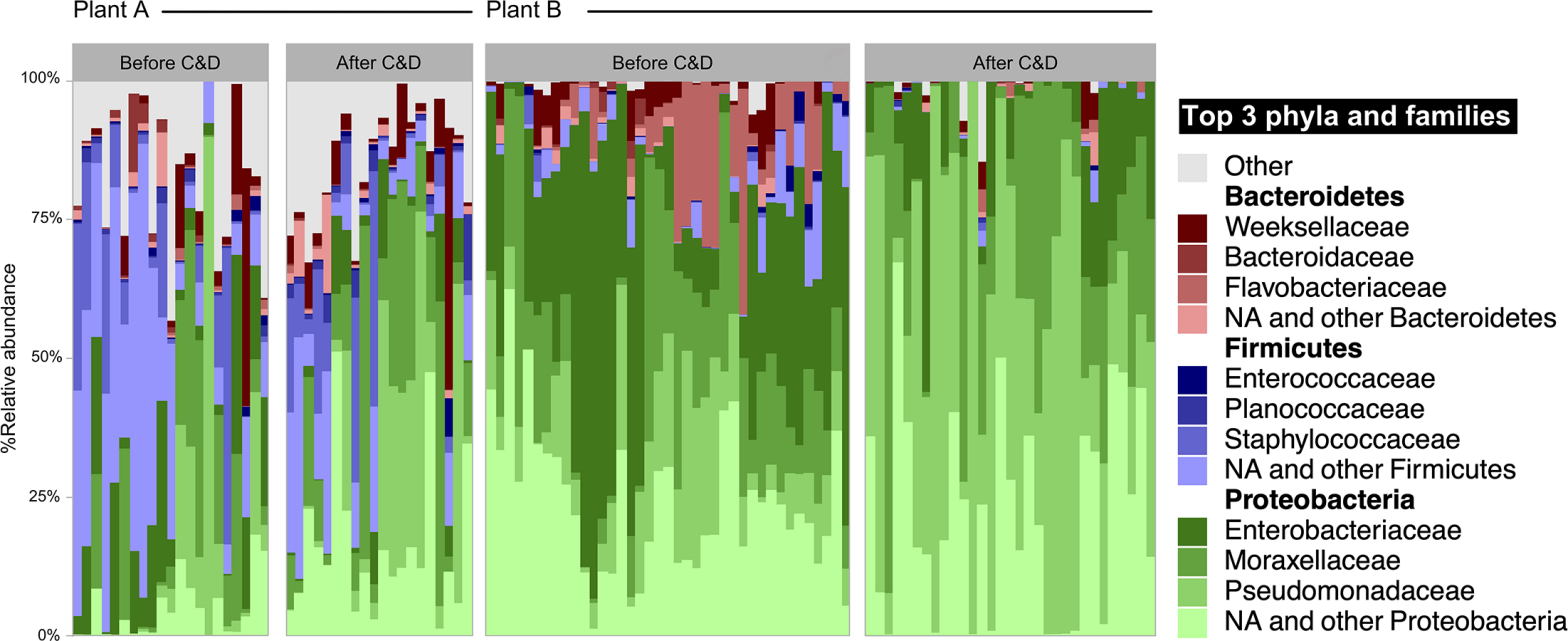
Top three phyla and families by % relative abundance before and after C&D in Plant A (*n* = 41) and Plant B (*n* = 70). The sum of all taxa outside of the top three phyla is marked “other.” Some of the sampling points are excluded due to insufficient data.

To further investigate shifts in the bacterial surface microbiota before and after C&D in broiler processing plants, we performed a metataxonomic analysis. The top three phyla by relative abundance were Proteobacteria (syn. Pseudomonadota), Firmicutes (syn. Bacillota), and Bacteroidetes (syn. Bacteroidota) (Fig. 5).

**Fig 6:**
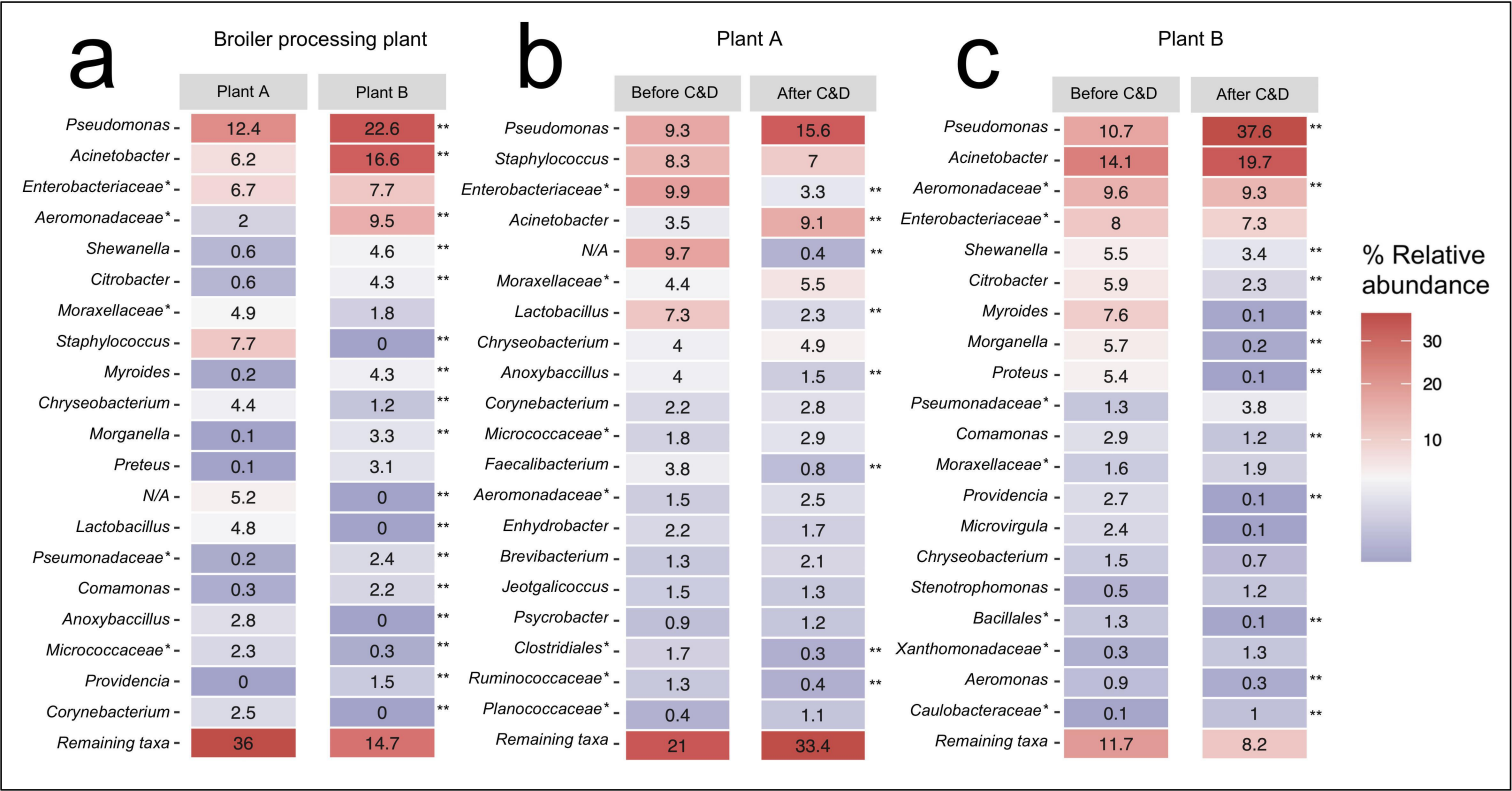
Top 20 bacterial genera by % mean relative abundance in Plants A and B (a) and before and after C&D (b, c). Taxa marked with a single asterisk (*) are classified to the family level, i.e., unknown genera. Numbers marked with a double asterisk (**) are significantly different by Wilcoxons signed-rank test (*P* < 0.05).

The mean relative abundance of Proteobacteria increased in both broiler processing plants after C&D from 37% to 54% in Plant A and from 83% to 97% in Plant B (Fig. A4). At the same time, Firmicutes decreased from 38% to 23% in Plant A and from 4.8% to 0.7% in Plant B. The most abundant bacterial genera in Plant A before C&D were an unknown genus of Enterobacteriaceae (10%), *Pseudomonas* (9%), and *Staphylococcus* (8%) (Fig. 6). After C&D, *Pseudomonas* was the most abundant genera (16%), while *Lactobacillus*, *Anoxybacillus*, *Faecalibacterium* decreased significantly, and *Acinetobacter* increased significantly (*P* < 0.05). In Plant B, the most abundant bacterial genera before C&D were *Acinetobacter* (14%), *Pseudomonas* (11%), and an unknown genus of *Aeromonadaceae* (10%). After C&D, *Pseudomonas* showed a significant increase, becoming the dominant genus (38%), while an unknown genus of Aeromonadaceae, *Shewanella*, and *Citrobacter* decreased significantly. Furthermore, we observed differences between genera found on FCSs compared with NFCSs in Plant A (Fig. A5). The relative abundance of *Pseudomonas* was only 0.3% on NFCSs and significantly higher on FCSs (17.4%). The most abundant genera on NFCSs were *Staphylococcus*, *Lactobacillus*, an unknown *Enterobacteriaceae* genus, and *Corynebacterium*. These had significantly lower relative abundance on FCSs, except for the unknown *Enterobacteriaceae*. While in Plant B, none of the top 20 genera were significantly different in relative abundance on FCSs compared with NFCSs.

**Fig 7:**
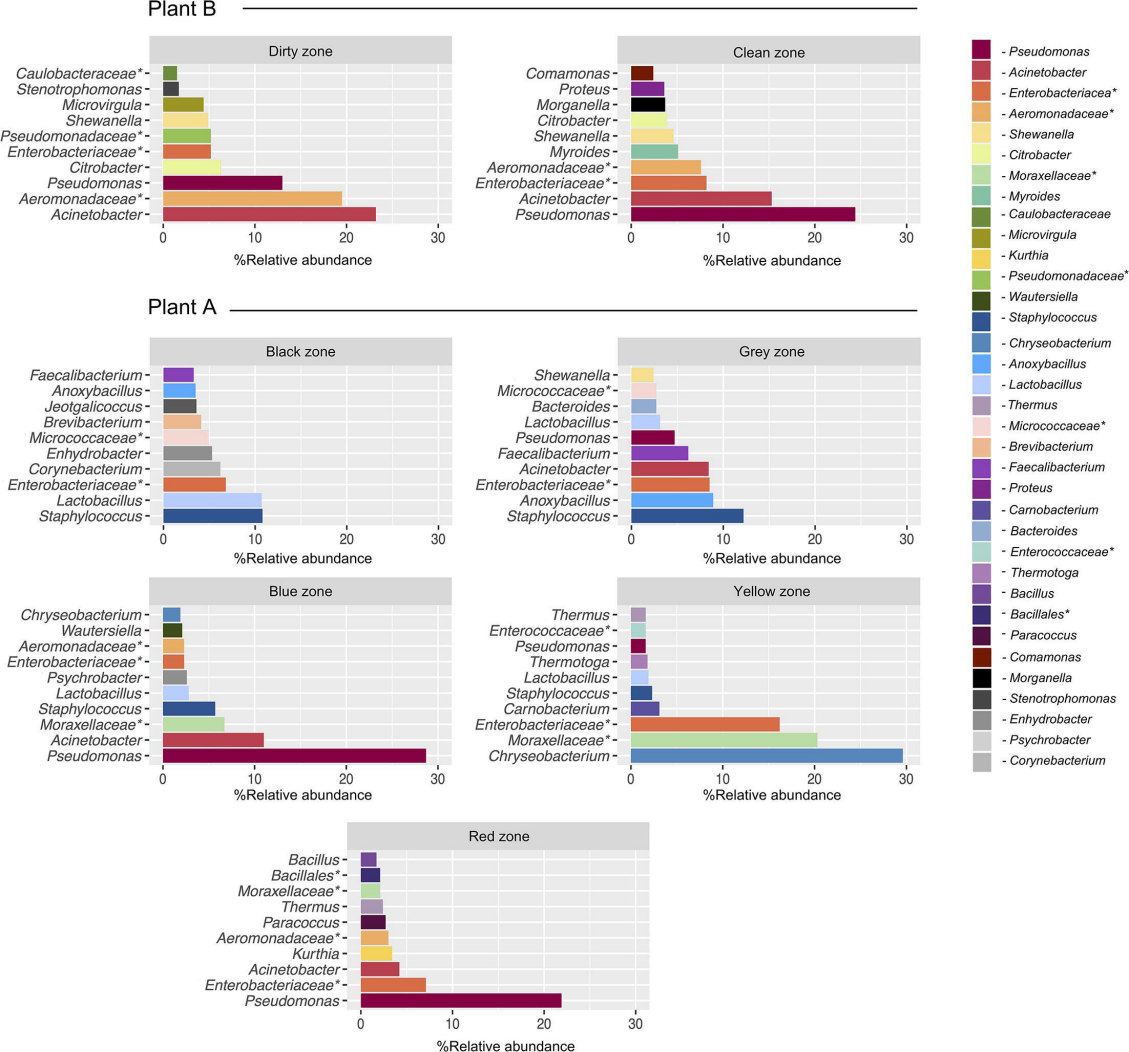
Horizontal bar plots showing the 10 most abundant bacterial genera in different hygienic zones, i.e., the dirty and clean zones in Plant A and the black, gray, blue, yellow, and red zones in Plant B. Taxa marked with an asterisk (*) are classified on family level, i.e., unknown genera.

The 10 most abundant bacterial genera varied depending on the hygienic zone in both processing plants (Fig. 7). Especially, the gray and black zones in Plant A varied from the other zones, likely due to contamination from broiler feathers, head, feet and gut. In these zones, *Staphylococcus*, *Lactobacillus*, *Anoxybacillus*, and an unknown Enterobacteriaceae genus were the most abundant. Genera, such as *Shewanella*, *Corynebacterium*, *Faecalibacterium*, and *Jeotgalicoccus*, were also among the 10 most abundant in the gray or black zone but not in the other zones. In the blue zone, *Pseudomonas*, *Acinetobacter*, and an unknown *Moraxellaceae* genus were the most abundant, in descending order. *Chryseobacterium* was the most abundant genus found in the yellow zone in the brining vessel in addition to unknown genera of Moraxellaceae and Enterobacteriaceae, and *Carnobacterium*. Heat-tolerant *Thermus* and *Bacillus* were among the top 10 genera in the red zone where heat-treated products are packaged (high-risk zone).

The most abundant genera in the red zone were *Pseudomonas*, an unknown Enterobacteriaceae genus, and *Acinetobacter*. In Plant B, differences between hygienic zones were smaller and six of the top ten taxa were found in both zones. In the dirty zone, *Acinetobacter*, an unknown *Aeromonadaceae* genus, and *Pseudomonas* were the most abundant, and *Pseudomonas*, *Acinetobacter*, and an unknown *Enterobacteriaceae* genus were the most abundant in the clean zone, in descending order. *Myroides*, *Morganella*, and *Proteus* were only found among the top 10 genera in the dirty zone. Finally, the mean relative abundance of the broiler relevant family *Campylobacteraceae*, including *Campylobacter* and *Acrobacter*, was negligible in both processing plants and overall, at less than 0.1%. Low abundances were found on the organ extraction unit and plucking machine in Plant A and on floors in the defeathering area in Plant B. Similarly, *Salmonella* had a mean relative abundance of less than 0.1% but was also found on the plucking machine in Plant A (0.3%).

**FIG 8:**
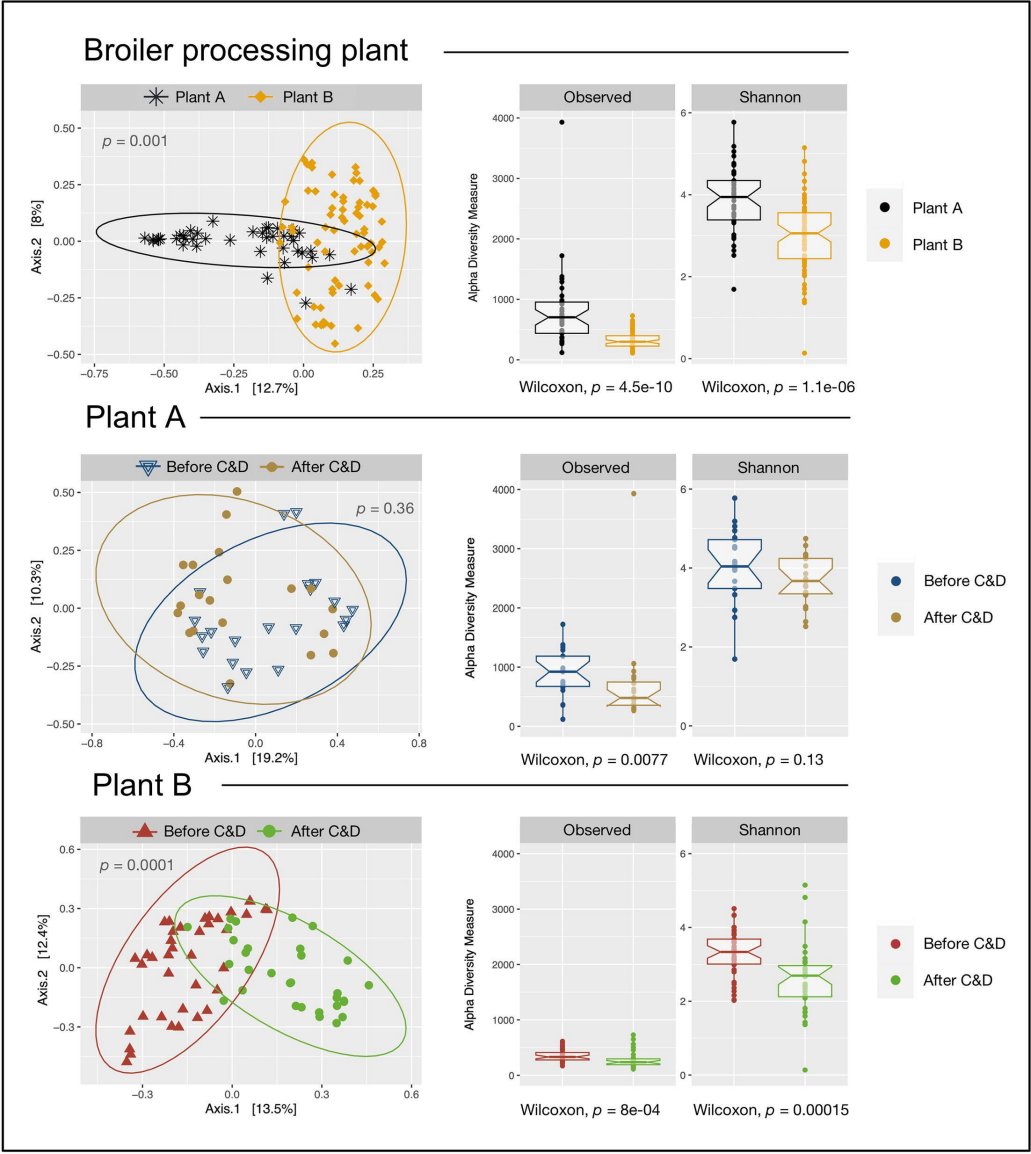
Alpha- and beta-diversity in samples from Plants A and B before and after C&D. Alpha-diversity is given by the observed richness and Shannon diversity index. Significant differences were calculated by Wilcoxon’s signed-rank test. Beta-diversity is shown by principal coordinates analysis (PCoA) plots with Bray-Curtis dissimilarities, and significant differences were analyzed using PERMANOVA (Adonis).

Significant differences were found in both alpha- and beta-diversity between Plants A and B (*P* < 0.01, Wilcoxon test and adonis) (Fig. 8). Both the observed richness and Shannon index were significantly higher in Plant A as compared with Plant B. Two significantly different clusters were observed for Plants A and B in the PCoA plots for beta-diversity (Bray–Curtis). In Plant B, the beta-diversity was also significantly different between samples taken before and after C&D. Both observed richness and Shannon index were significantly lower in samples taken after C&D. The same was not observed in Plant A, where both the beta-diversity and Shannon index did not differ significantly before and after C&D. Yet, the observed richness was significantly lower after C&D in Plant A. Moreover, we compared the alpha- and beta-diversity in samples from FCSs and NFCSs, warm season, cold season, and different hygienic zones (Fig. A6 to A8). Significant differences in both the alpha- and beta-diversity measures were found between the warm and cold seasons in Plant B. In Plant A, the Shannon index was significantly lower in the yellow zone compared with the black, blue, and gray zones. Altogether, we found that C&D affected both the relative abundance and diversity of the bacterial surface microbiota found in broiler processing plants.

### Most of the minimum inhibitory concentrations were below the highest concentration recommended by the producer of commercial disinfectants

Minimum inhibitory concentration (MIC) testing of commercial DIs with bacterial isolates from Plant A (*n* = 175) and Plant B (*n* = 138) revealed that five out of seven DIs inhibited all isolates at the highest recommended concentration by the producer (Table 3). These include Aqua DES Foam, Enduro Timesaver, Calgonit DS 680, Delladet VS2, and Aqua San AM Plus. The latter two inhibited all isolates at the lowest concentration tested. Interestingly, Aqua DES Foam and Enduro Timesaver inhibited most isolates at the lowest concentration tested, yet Enterobacterales isolates from Plant A seemed to tolerate a higher concentration of these DIs compared with the remaining isolates. Only 44% or less of these were inhibited at 0.5% Aqua DES Foam and 2% Enduro Timesaver compared with more than 80% of the remaining isolates. Most of the Enterobacterales isolates were *E. coli* strains (Table A6). At 4% of Enduro Timesaver, both Enterobacterales and *Pseudomonas* isolates were completely inhibited. While complete inhibition of all isolates was achieved at 1% of Calgonit DS 680, no differences were observed between Enterobacterales isolates from Plants A and Plant B. Calgonit DS 680 inhibited more of the Enterobacterales isolates (80%) compared with the *Pseudomonas* isolates (45%) at a concentration of 0.015%. Altogether, these DIs were effective to inhibit the bacterial isolates even below the recommended user concentration.

**TABLE 3:** Distribution of MIC values of eight DIs for 313 isolates sampled before and after C&D in Plants A and B divided into *Pseudomonas* (PSE) and Enterobacterales (ENT)

Disinfectant	Conc. %	Distribution (%) of MIC values					
		Plant A		Plant B		Isolates sampled before or after C&D	
		PSE *n* = 103	ENT *n* = 72	PSE *n* = 42	ENT *n* = 96	Before *n* = 215	After *n* = 98
Aqua Des Foam PAA	0.5	100	44	100	97	81	99
	1.0	0	56	0	3	19	1
	2.0^*a*^	0	0	0	0	0	0
							
Delladet VS2	0.9	100	100	100	100	100	100
	1.8^*a*^	0	0	0	0	0	0
	3.6	0	0	0	0	0	0
							
Titan Hypo	1.0^*a*^	1	41	38	76	43	22
	5.0	93	59	63	24	56	72
	7.5	5	0	0	0	0	6
	>7.5	1	0	0	0	1	0
							
Aqua Biocip	0.3	0	0	16	4	4	0
	2.0^*a*^	94	96	84	96	94	94
	5.0	6	4	0	0	2	6
							
Aqua San AM Plus	1.0	100	100	100	100	100	100
	3.0^*a*^	0	0	0	0	0	0
	5.0	0	0	0	0	0	0
							
Enduro Timesaver	2.0	81	39	100	83	70	80
	4.0	19	61	0	17	30	20
	8.0^*a*^	0	0	0	0	0	0
							
Calgonit DS 680	0.015	45	80	44	79	72	45
	1.0	55	20	56	21	28	55
	3.0^*a*^	0	0	0	0	0	0
							
Ecas4	4	0	0	10	1	1	1
	50	6	7	16	9	9	7
	80	88	93	74	90	88	90
	>80	6	0	0	0	2	2

^*a*^ Highest user concentration recommended by the producer.

Two of the seven tested DIs, Titan Hypo and Aqua Biocip, did not inhibit all isolates at the recommended user concentration. These were the only DIs based on sodium hypochlorite in the selection. The efficacy of the recommended user concentration of Ecas4 (100%) was not determined in this study. The highest concentration tested (80%) inhibited all isolates except 6% of the *Pseudomonas* isolates from Plant A. Titan Hypo inhibited only 1% of the *Pseudomonas* isolates from Plant A at the recommended concentration (1%) and around half of the Enterobacterales isolates (41%). At 5% of Titan Hypo, 94% and 100% of *Pseudomonas* and Enterobacterales isolates were inhibited in addition to all the isolates from Plant B. The remaining isolates were inhibited at 7.5% except one isolate (*P. fluorescens*). When comparing MIC values of isolates sampled before and after C&D, we observed tendencies that isolates sampled after C&D had higher MIC values for several DIs. For example, 43% of the isolates sampled before C&D were inhibited at 1% of Titan Hypo compared with 22% of those sampled after C&D. Similar results were also observed for Calgonit DS 680, while the differences were smaller for Ecas4 and Aqua Biocip where only a few of the isolates sampled after C&D had higher MICs. In contrast, Enduro Timesaver and Aqua Des Foam showed higher MIC values among isolates sampled before C&D, suggesting that isolates sampled after C&D do not always tolerate higher concentrations of all the DIs. Furthermore, > 80% of the MIC values for Aqua Biocip were at the recommended user concentration (2%). All isolates were inhibited at a concentration of 5%, and differences between MIC values for Enterobacterales and *Pseudomonas* from Plants A and B were small. As anticipated, our findings demonstrate that the effectiveness of DIs is closely tied to their concentration levels.

### Biofilm luminescence was reduced significantly after 20 min exposure towards different commercial disinfectants

**Fig 9:**
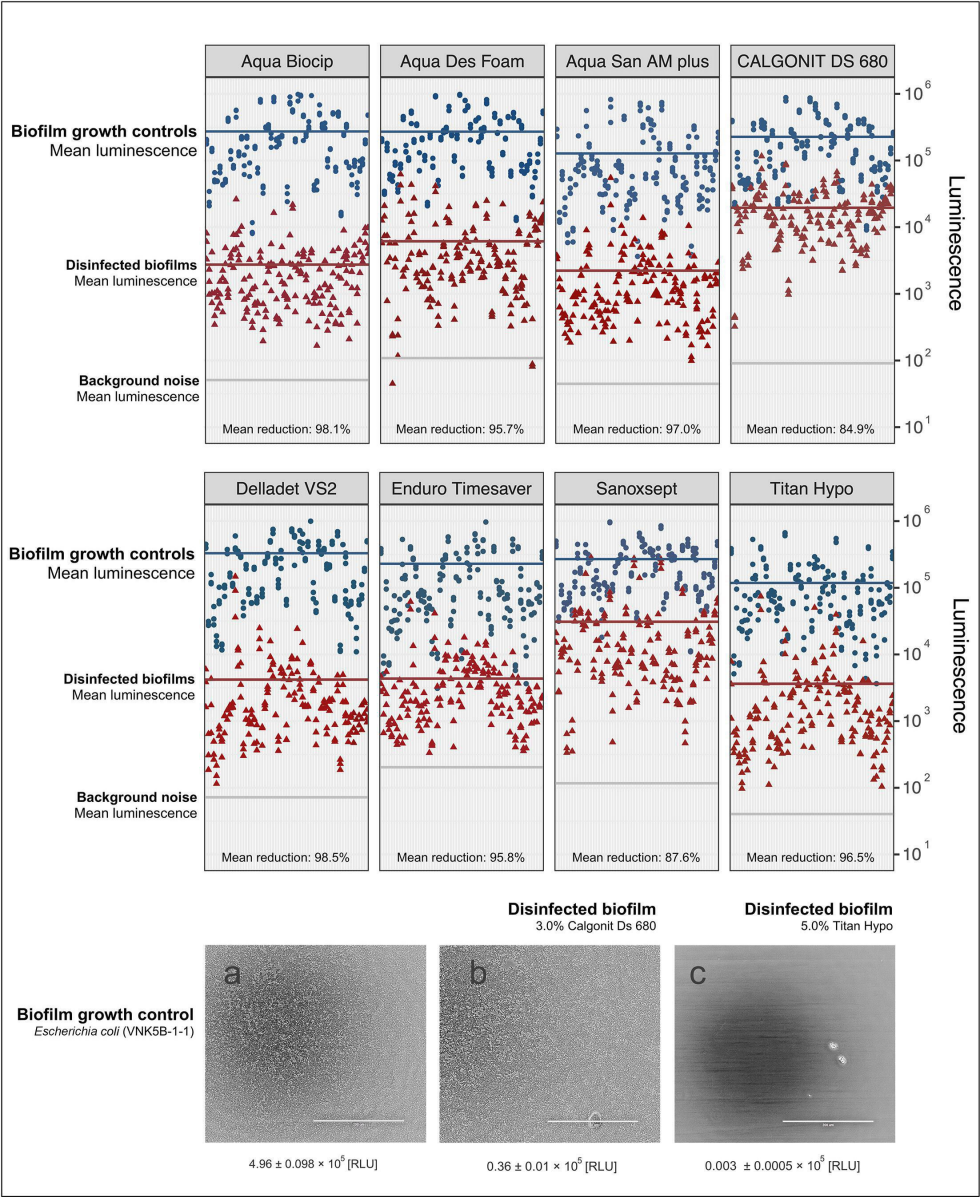
Dot plots comparing luminescence in biofilm growth controls (blue dots) and biofilms after 20 min exposure to DIs at their highest recommended user concentration (red triangles). Each group includes 62 biofilms in triplicates per DI formed by *Pseudomonas*, Enterobacterales, *Acinetobacter*, *Aeromonas*, and *Enterococcus*. Background noise representing luminescence in negative controls is included to show that disinfected biofilms near this level indicate high DI efficacy. Horizontal plot lines show the mean luminescence. Sectional micrographs show examples of a *E. coli* biofilm growth control (**A**) and parallel biofilms after 20 min exposure to 3% Calgonit DS 680 (**B**) and 5% Titan Hypo (**C**). Additional micrographs found in Fig. A10 to A14.

We performed an HTS test with 62 biofilms, measuring viability with BacTiter GLO after 20 min of exposure to eight DIs. At the highest recommended user concentrations, all eight DIs significantly reduced the mean luminescence (RLU) in biofilms as compared with biofilm growth controls (*P* < 0.01) (Fig. 9). For example, the mean RLU in biofilms disinfected by Aqua Biocip was 2.7 × 10^3^ and significantly lower than the mean RLU in the respective biofilm growth controls (2.7 × 10^5^). Yet, the mean RLU in disinfected biofilms was still more than 10-fold higher than the background noise (5.1 × 10^1^), indicating that some of the biofilms survived the exposure.

**Fig 10:**
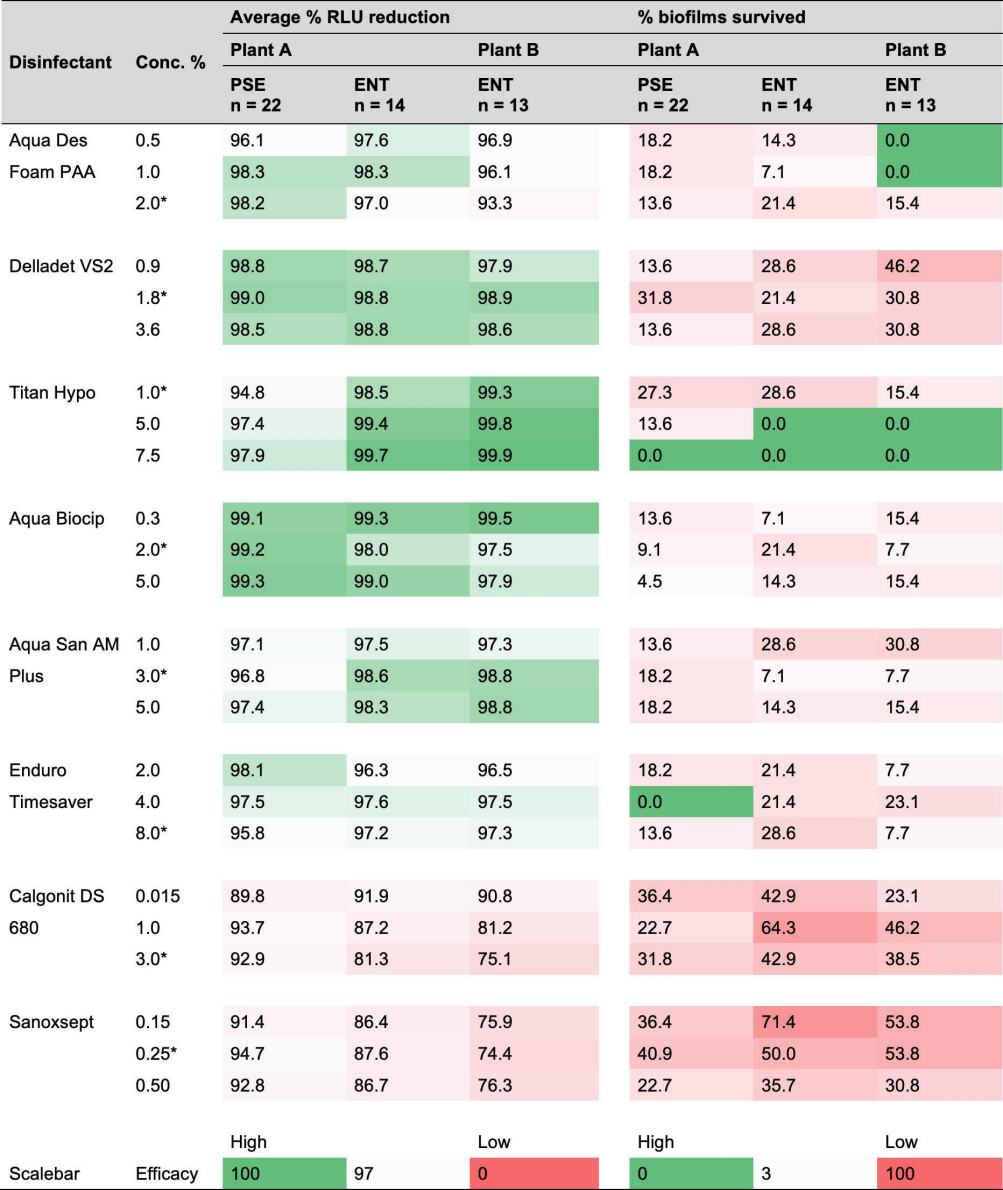
Average percent reduction of luminescence (RLU) in biofilms after 20 min exposure to three concentrations of eight disinfectants. Biofilms are grouped into *Pseudomonas* (PSE) and Enterobacterales (ENT) from Plants A and B. The right side of the figure shows the percentage of biofilms that survived. Green to red color indicates the relative efficacy of the disinfectant against biofilms at the given concentration from high to low. The highest recommended user concentration by the producer is marked with an asterisk (*).

Generally, large RLU variations were observed depending on the species and isolate that formed the biofilm as the selection included *Pseudomonas*, Enterobacterales, *Acinetobacter*, *Aeromonas*, and *Enterococcus* isolates. A direct comparison between the RLU of biofilms formed by different species or strains may be inappropriate due to natural variations in the ATP content of different species-specific cells. Therefore, we calculated the RLU reduction ratio (%) to better understand differences between DIs, different concentrations of these, and different bacterial species. For example, the RLU (× 10^5^) in a biofilm growth control formed by *E. coli* was reduced from 4.96 to 0.36 in the disinfected biofilm by 1% Calgonit DS 680, resulting in a 93% reduction (Fig. 9a and b). Overall, the mean reduction by Sanoxsept (88%) and Calgonit DS 680 (85%) were significantly lower than the mean reduction by other DIs (> 95%) at the recommended concentration (Tukey post-hoc test, *P* < 0.05). These results suggest that these DIs were less effective than the others, as the highest overall mean reduction, 99%, was achieved by Delladet VS2. (Fig. A9). Only small differences were found between the mean reduction in biofilms formed by different species. Comparing *Pseudomonas* and Enterobacterales biofilms from Plant A, differences in mean reductions were only 1%–2% by disinfectants, such as Aqua Des Foam, Delladet VS2, Enduro Timesaver, Aqua Biocip, and Aqua San Am Plus (Fig. 10), regardless of the concentration. Although Titan Hypo (1%) seemed to be less effective against *Pseudomonas* biofilms (mean reduction: 95%) than Enterobacterales biofilms (mean reduction: 99%) from Plant A. Interestingly, the same was observed during the MIC test, where *Pseudomonas* had higher MICs than Enterobacterales. Furthermore, Enterobacterales biofilms formed by isolates sampled in Plant A seemed to tolerate less than those from Plant B. At the recommended concentration, four out of eight DIs resulted in larger mean reductions in Enterbacterales biofilms from Plant A. The differences were negligible for three of the DIs (<0.2%). Contrary to the MIC test, the efficacy of the DIs was depending less on the different concentrations tested when comparing the mean RLU reductions. Higher concentrations resulted in larger reductions for some DIs, but the differences were overall small.

To investigate how many of the biofilms survived the exposure to the DIs, we defined survival as mean RLU in exposed biofilms 10 times greater and significantly higher than mean RLU in the background noise (*P* < 0.01). This ensures the presence of a robust signal that is likely to represent a substantial presence of viable biofilm and accounts for experimental variation. Only one concentration of one DI (7.5% Titan Hypo) eradicated all *Pseudomonas* and Enterobacterales biofilms (Fig. 10). At the recommended user concentration, none of the DIs eradicated all biofilms, and the survival was between 7% and 53% depending on the DI. Aqua Des Foam, Aqua Biocip, and Aqua San Am Plus were most effective at this concentration and only 7%–22% of the biofilms survived. Equally to the mean RLU reductions, Sanoxsept and Calgonit DS 680 were less effective and 22%–72% survived depending on the concentration and bacterial group. Ultimately, our results show that biofilms are significantly reduced by different DIs, although they are likely surviving disinfection and may re-grow under the correct conditions.

## DISCUSSION

The surface microbiota in broiler processing plants can be a source of contaminating bacteria, affecting both the safety and spoilage of broiler meat. Our results show that C&D significantly reduced the average bacterial loads from 3.7 to 1.8 log CFU/cm^2^ in Plant A. Before C&D, the most abundant bacterial genera were an unknown genus of *Enterobacteriaceae* (10%), *Pseudomonas* (9%), and *Staphylococcus* (8%). After C&D, *Pseudomonas* became the most abundant genus (16%), while *Lactobacillus*, *Anoxybacillus*, and *Faecalibacterium* decreased significantly, and *Acinetobacter* increased significantly. C&D reduced alpha-diversity with a significant reduction in observed richness, but beta-diversity was not significantly affected. Furthermore, bacterial loads declined across the five hygienic zones along the chicken processing line. In the black zone, where broilers are slaughtered, the bacterial microbiota was dominated by *Staphylococcus*, *Lactobacillus*, *Anoxybacillus*, and bacterial loads were significantly higher than in the blue post-slaughter zone which was dominated by *Pseudomonas*.

In Plant B, C&D significantly reduced the average bacterial loads from 7.0 to 3.8 log CFU/cm^2^. Before C&D, the most abundant bacterial genera were *Acinetobacter* (14%), *Pseudomonas* (11%), and an unknown genus of *Aeromonadaceae* (10%). After C&D, *Pseudomonas* increased significantly and became the most abundant genus (38%), while an unknown genus of *Aeromonadaceae*, *Shewanella*, and *Citrobacter* decreased significantly. C&D significantly reduced both alpha- and beta-diversity. *Acinetobacter* and an unknown genus of *Aeromonadaceae* dominated the dirty zone, while *Pseudomonas* was the most abundant in the clean zone. No significant differences were observed in the bacterial loads between zones. At the recommended user concentrations, the DIs effectively inhibited planktonic bacteria and significantly reduced mono-species biofilms from both plants. However, none of the DIs completely eradicated all biofilms at these concentrations, with survival rates ranging from 7% to 53%, depending on the DI.

Numerous studies have investigated total aerobic counts on broiler carcasses during processing (37–39). Nevertheless, studies focusing on surfaces in broiler processing plants are limited. Kim and Yim (40) investigated sanitized surfaces, such as cutting machines, worktables, boots, and aprons in beef and pork processing plants and found total aerobic counts between 1.69 to 3.87 log CFU/cm^2^. These results are similar to our bacterial counts, which were on average, 1.8 and 3.8 log CFU/cm^2^ after C&D in Plants A and B, respectively. In our previous study, we found that bacterial loads were approximately 2.0 log CFU/cm^2^ on surfaces sampled after C&D in a salmon processing plant (23). Moreover, we observed that bacterial loads declined from the start to end of the broiler processing line across hygienic zones in both processing plants with significant differences in Plant A. Similar results have been found in studies investigating broiler carcasses. Hauge et al. (37) analyzed total plate counts on broiler carcasses before scalding, after plucking, after evisceration, and after chilling. The authors found that counts decreased significantly along the broiler processing line with largest reductions after scalding, which can be explained by high water temperatures of 50°C–60°C during scalding (41). In our study, the largest reductions in bacterial counts were observed between the black, gray, and blue zones in Plant A. While counts were high in both the black and gray zones (5.0 and 2.9 log CFU/cm^2^), likely due to the presence of broiler feces, feathers, and intestines, they were significantly lower in the blue zone (1.5 log CFU/cm^2^), where chilled carcasses are trimmed, deboned, sized, and packaged. This suggests that hygienic zones are controlling the spread of bacterial contaminants from slaughtering stages where high levels of bacteria were found. Additionally, bacterial loads decreased in all the hygienic zones after C&D in both processing plants, with significant differences in several of these zones. Log reductions were almost two log units on average in Plant A and more than three log units in Plants B. Although we could not find comparable studies on this, the reductions were higher than those we observed in our previous study (23). In the salmon processing plant, C&D reduced the bacterial loads by an average of 1.1 log CFU/cm^2^.

Despite significant log reductions, we isolated presumptive *Pseudomonas* spp. on >80% of the surfaces in Plant A after C&D. The high prevalence of *Pseudomonas* was also confirmed by metataxonomics, showing that *Pseudomonas* had the highest relative abundance after C&D in both processing plants. Similar results were found by Marmion et al. (42) where *Pseudomonas* was quantified on contact surfaces, such as defeathering machines, evisceration equipment, conveyor belts, and filleting blades in Irish poultry processing plants using selective media. The authors detected high levels of *Pseudomonas* spp. on all surfaces both before production started and midway production, while the abundance of *Campylobacter*, *Salmonella*, and *Enterobacteriaceae* was generally lower. Nevertheless, the authors did not perform species-level identification of isolates. We found that most of the *Pseudomonas* spp. isolated from Plant A belonged to the *P. fluorescens* group in subgroups of *P. fluorescens*, *P. fragi*, *P. koreensis*, and *P. gessardii*. Several of these species have previously been shown to grow at refrigeration temperatures (4°C) and to produce spoilage associated enzymes and pigments (43). For example, Circella et al. (44) demonstrated that a member of the *P. fluorescens* group can be responsible for the blue coloration of meat. Although members of the group are not considered dangerous to humans, signs of spoilage, such as coloration, off-odor, and off-taste affect consumer perception and can contribute to food waste and economic loss. *Salmonella* and *Campylobacter* are typically associated with broiler meat (3), yet our culture independent analyses showed negligible abundances of these in the processing environment. Moreover, high relative abundances of *Acinetobacter* and an unknown genus of *Enterobacteriaceae* were found in both processing plants. A high relative abundance of *Staphylococcus* was only found in Plant A, while an unknown genus of *Aeromonadaceae* and *Shewanella* were especially abundant in Plant B. Marmion et al. (42) also found high relative abundances of genera, such as *Aeromonas*, *Shewanella*, and *Acinetobacter* in Irish poultry processing plants, whereas Song et al. (45) found high relative abundances of *Curvibacter*, *Sphingomonas*, and *Acinetobacter* on conveyor belts and electronic scales in Chinese poultry processing plants during production. This suggests that bacterial communities vary depending on the processing plant, with some similarities.

Interestingly, we also found differences between different hygienic zones within processing plants. In the black and gray zones in Plant A, where broilers are slaughtered and eviscerated, the bacterial microbiota was dominated by *Staphylococcus*, *Lactobacillus*, *Anoxybacillus*, and an unknown genus of *Enterobacteriaceae*. Some of these are likely originating from broiler intestines, feces*,* and feathers. For example, Richards-Rios et al. (46) found high abundances of *Lactobacillus* and *Escherichia* in the ileal mucus and lumen of different broilers. A study investigating bacterial communities on broiler carcasses found a major shift in the relative abundance of *Staphylococcus* from 26% pre-scalding to <2% post-scalding (39). Similarly, our results showed that *Staphylococcus* only dominated the black and gray zones, while their relative abundance dropped in the remaining post-slaughter zones. In the blue and red zones, *Pseudomonas* was predominant, while *Chryseobacterium* showed the highest relative abundance in the yellow zone. These bacteria have previously been described as halotolerant (47), which may explain their dominance in this zone since we only sampled the brining vessel. Altogether, our findings demonstrate that the bacterial surface microbiota is primarily dominated by potential spoilage bacteria such as *Pseudomonas*. However, we also isolated bacteria which can affect the safety of broiler meat, including *L. monocytogenes, E. coli, Y. enterocolitica, A. baumannii*, and *P. aeruginosa*. While most of these potential pathogens were detected before C&D, their presence highlights the importance of effective cleaning routines and reliable DIs.

Most of the DIs we tested were effective at the highest recommended concentration against planktonic bacteria. The results were similar to our previous study where isolates sampled in a salmon processing plant were tested against the same DIs (23). Differences were observed between Enterobacterales isolates, for example, >98% of Enterobacterales from the salmon processing plant were inhibited at the lowest concentration tested by Aqua Des Foam, Titan Hypo, and Enduro Timesaver. However, <44% of Enterobacterales from Plant A were inhibited at this concentration by the same DIs. This may be explained by differences in DI tolerance between genera, since *Hafnia* spp. were the most identified Enterobacterales in the salmon processing plant, opposed to *Escherichia* spp. in Plant A. Moreover, we found that all the tested DIs significantly reduced the mean luminescence (RLU) in biofilms at the highest recommended concentrations. Yet, none of the DIs eradicated all biofilms at this concentration and the survival was between 7% and 53% depending on the DI. Although comparable studies investigating the same DIs are limited, a few studies have shown that DIs based on hydrogen peroxide are more effective against biofilms compared with DIs based on QACs (19, 48). This aligns with our results, showing that more biofilms survived exposure to QAC-based DIs like Calgonit DS 680 and Delladet VS2 compared with Aqua Des Foam, which is based on hydrogen peroxide and peracetic acid. However, Sanoxsept deviates from this trend, as it is also hydrogen peroxide-based, yet many biofilms survived the exposure. This could be explained by the recommended concentration of this DI, as the active component levels may differ from those in Aqua Des Foam.

Since the efficacy of DIs depends on both concentration and contact time (11), the MIC results should be interpreted with caution. In practice, the exposure time is usually 15–30 min, while MIC methods typically involve 24 h incubation. The biofilm experiments we performed are, however, reflecting a more realistic scenario where DIs are applied for 20 min and rinsed off afterwards. While optical density measurements have a detection limit of approximately 10^7^ CFU/mL (49), BacTiter-Glo enabled us to detect much lower levels of viable cells, i.e., the method is 1,000-fold more sensitive than absorbance readings (50). A study by Stiefel et al. (51) described this method as precise with a broad detection range; however, the authors corroborated that BacTiter-Glo only gives an answer to the viability of cells and not whether the biofilm matrix itself is removed (EPS layers, i.e., extracellular polymeric substances). Future studies may therefore combine several methods to more accurately determine both biofilm eradication and removal. An additional limitation of this study is connected to the presence of DNA from dead cells in the processing environment, introducing bias to the sequencing analyses. Especially after C&D, the observed relative abundances may not accurately reflect the viable surface microbiota. RNA sequencing may give more accurate results. For example, Li et al. (52) performed both DNA and RNA sequencing and found discrepancies between the relative abundances of bacterial taxa with RNA sequencing being superior in accuracy. Nevertheless, combining the sequencing analyses with culture-dependent methods reinforces our findings for certain bacterial taxa and their viability both before and after C&D.

To conclude, this study provides evidence that the bacterial surface microbiota in broiler processing plants is a source of potential spoilage bacteria and also some bacteria associated with pathogenicity. C&D effectively reduces the bacterial burden but also reshapes the bacterial microbiota favoring certain taxa like *Pseudomonas*. Moreover, the bacterial community structure changes depending on hygienic zones. A processing environment with more than two hygienic zones has shown to be advantageous with respect to bacterial transmission from slaughter to post-scalding processing. These insights highlight the need for targeted C&D routines considering zone-specific challenges. Plant operators should also be aware that biofilms can survive the exposure to disinfectants and re-grow under favorable conditions. This can facilitate the persistence of certain bacteria in these environments and increase the risk of food contamination and foodborne disease outbreaks. This suggests that there is a need for DIs with stronger biofilm eradication capabilities potentially in combination with mechanical cleaning. Ultimately, our study can contribute to understand and improve the state of hygiene in broiler processing plants.

## Data Availability

The raw sequencing reads used in the metataxonomic analysis from this study have been deposited in the NCBI Sequence Read Archive under BioProject, accession PRJNA1213099. Sanger sequences of isolates are found in Table A1.

## SUPPLEMENTAL MATERIAL

Supplemental figures aem.02401-24-s0001.pdf Figures A1 to A14.

Supplemental tables aem.02401-24-s0002.xlsx Tables A1 to A6.

ASM does not own the copyrights to Supplemental Material that may be linked to, or accessed through, an article. The authors have granted ASM a non-exclusive, world-wide license to publish the Supplemental Material files. Please contact the corresponding author directly for reuse.
