# Supplementary material for: Shifts in surface microbiota after cleaning and disinfection in broiler processing plants: incomplete biofilm eradication revealed by robotic high-throughput screening
Source: Appl Environ Microbiol. 2025 Feb 26;91(3):e02401-24. doi: 10.1128/aem.02401-24 (PMC11921370; doi:10.1128/aem.02401-24)
Supplement: Supplemental figures — Figures A1 to A14. [file aem.02401-24-s0001.pdf]

AMERICAN SOCIETY FOR MICROBIOLOGY | Applied and Environmental Microbiology

journal homepage: https://journals.asm.org/journal/aem

Shifts in surface microbiota after cleaning and disinfection in broiler processing plants; incomplete biofilm eradication revealed by robotic high-throughput screening

Thorben Reiche [a, *], Gunhild Hageskal [b], Mihai Mares [c], Sunniva Hoel [a], Anne Tøndervik [b], Tonje Marita Bjerkan Heggeset [b], Tone Haugen [b], Sigri Bakken Sperstad [b] Hanne Hein Trøen [b], Solfrid Bjørkøy [d], Anita Nordeng Jakobsen [a]

[a] *Department of Biotechnology and Food Science, Norwegian University of Science and Technology, Trondheim, Norway*
[b] *Department of Biotechnology and Nanomedicine, SINTEF Indsustry, Trondheim, Norway*
[c] *Department of Publich Health, "Ion Ionescu de la Brad" University of Life Sciences, Iași, Romania*
[d] *Norsk Kylling, Havneveien 43, 7300, Orkanger, Norway*
[*] *Corresponding author. E-mail address: thorben.reiche@ntnu.no (T. Reiche)*

Supplemental material: Figures

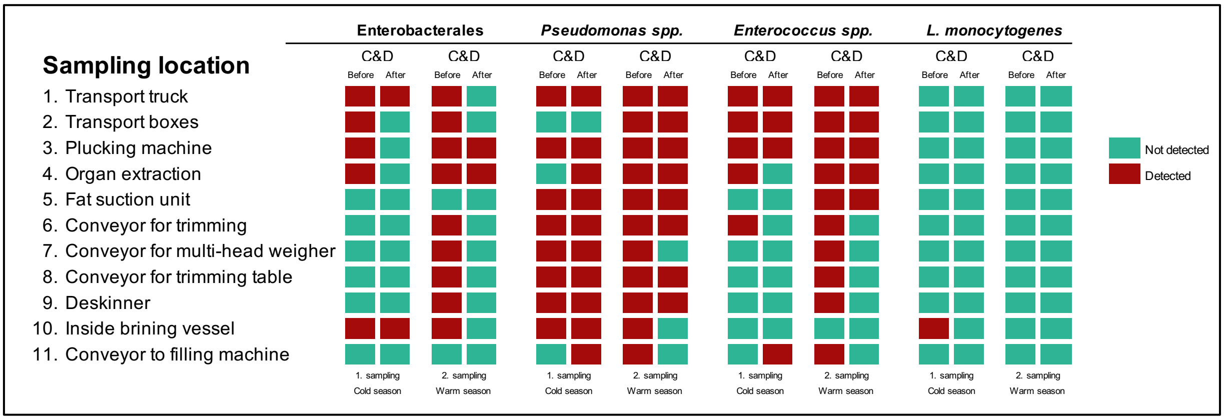

Fig. A1. Prevalence of presumptive bacterial taxa in a broiler processing plant (Plant A) divided into Enterobacterales, *Pseudomonas* spp., *Enterococcus* spp. and *L. monocytogenes* at sampling points (1-11) before and after C&D. Sampling was performed once during the cold and warm season. The results are based on growth or no growth on the selective media VRBGA, *Pseudomonas* CFC and Slanetz & Bartley. While detection of *Listeria* species and *Listeria monocytogene*s was performed using the SureTectTM *Listeria* species PCR Assay and *Listeria monocytogenes* PCR. Red color indicates detection, while green indicates no detection.

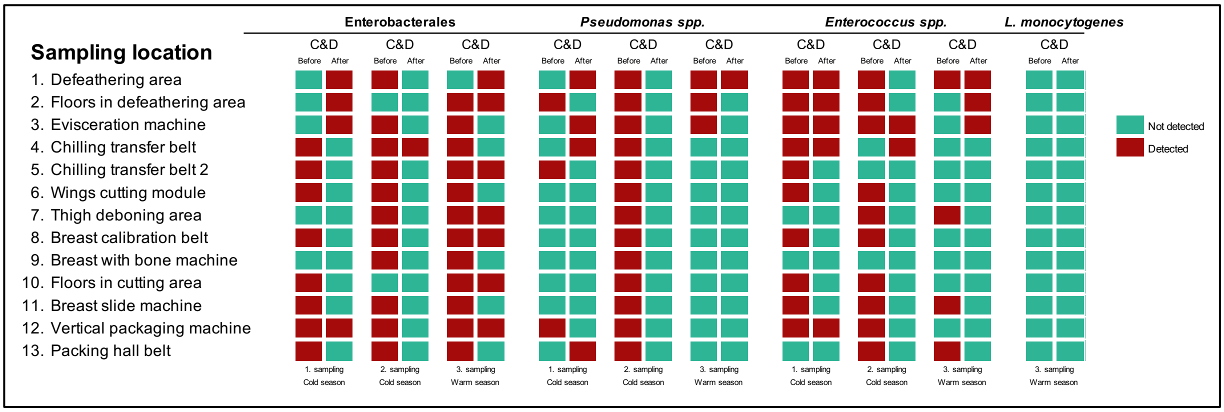

Fig. A2. Prevalence of presumptive bacterial taxa in a broiler processing plant (Plant B) divided into Enterobacterales, *Pseudomonas* spp., *Enterococcus* spp. and *L. monocytogenes* at sampling points (1-13) before and after C&D during the cold and warm season. The results are based on growth or no growth on the selective media RAPID'E.coli 2, RAPID'P.aeruginosa and RAPID'Enterococcus. While detection of *Listeria* species and *Listeria monocytogene*s was performed using the SureTectTM *Listeria* species PCR Assay and *Listeria monocytogenes* PCR. Red color indicates detection, while green indicates no detection. Sampling was performed twice during winter and once during summer. The prevalence of *L. monocytogenes* was only analyzed during the summer sampling. One sampling point (sampling point 14. Packing hall belt 2) was excluded because only a limited amount of data was obtained, i.e., the sampling point was only included in one of the three samplings.

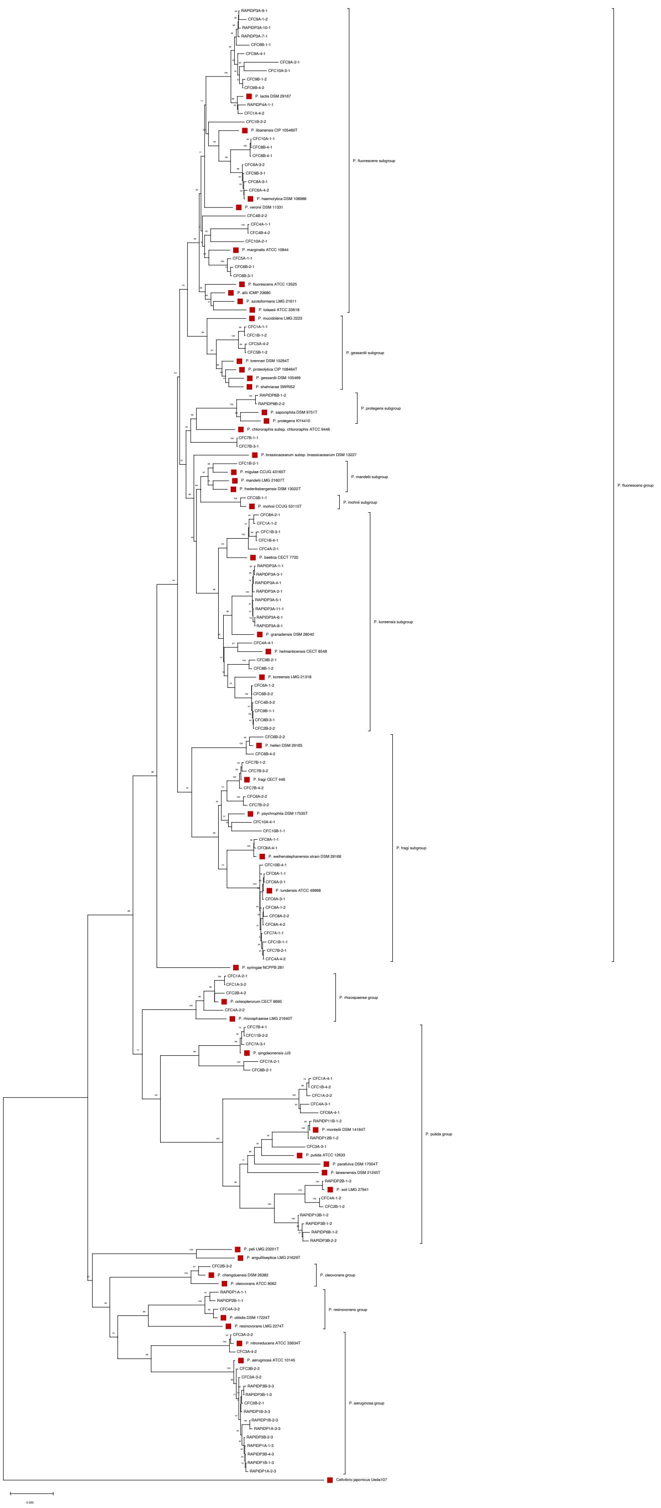

Fig. A3. Neighbor-joining tree based on *rpoD*-sequences of *Pseudomonas* spp. isolated from Plant A (n = 91) and Plant B (n = 33). Isolates from Plant A were sampled by CFC Pseudomonas agar, while isolates from Plant B were sampled by RAPID'P.aeruginosa. Taxa are divided in groups (G) and subgroups (SG) based on 49 reference strains (marked in red) belonging to these groups and obtained from the GenBank database.

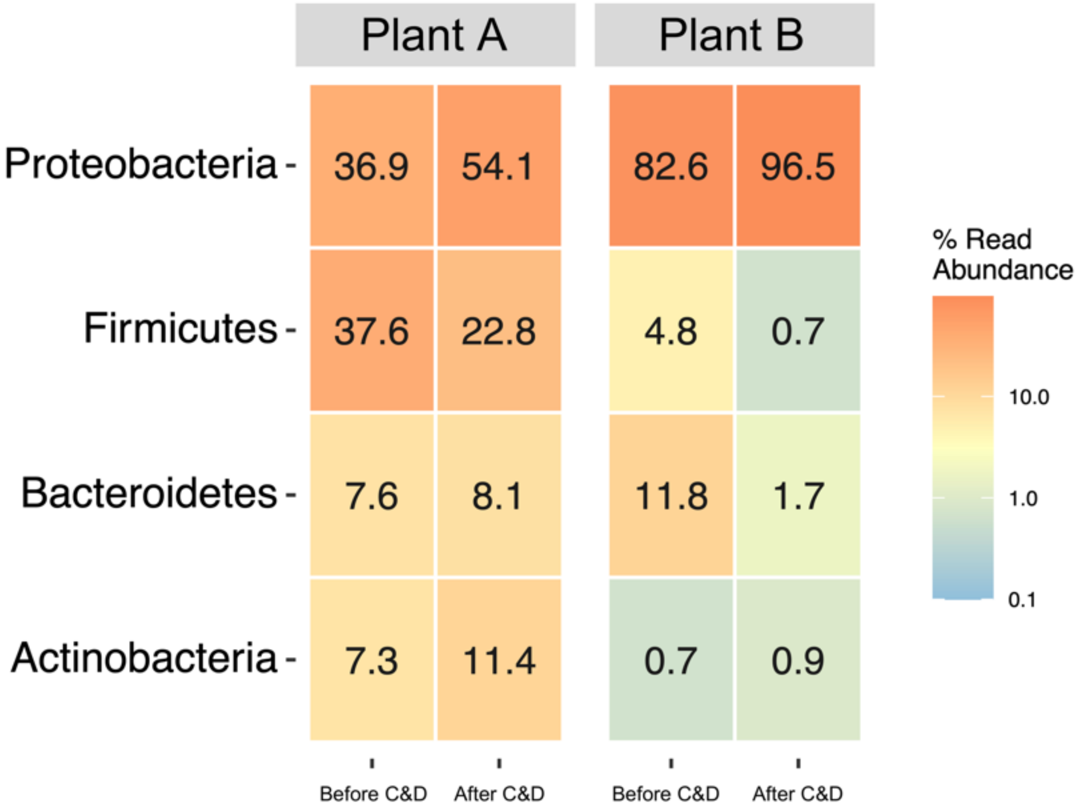

Fig. A4. Heatmap of top four bacterial phyla with highest % mean relative abundance before and after C&D in Plant A and Plant B.

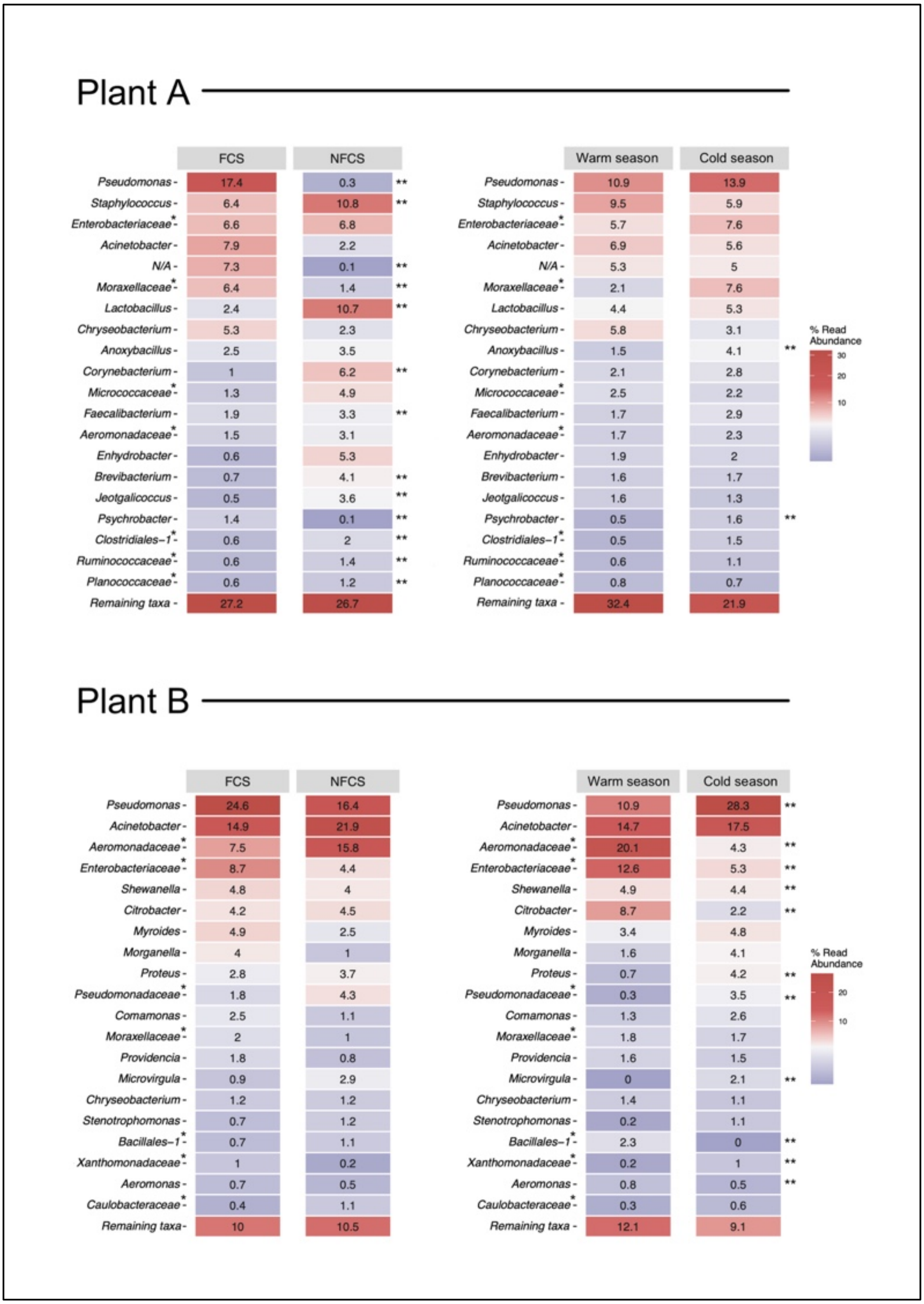

Fig. A5. Heatmap of 20 bacterial genera with highest relative abundance on FCSs (food contact surfaces) and NFCSs (non-food contact surfaces) and comparison between samplings during the cold and warm season at Plant A and Plant B. Taxa marked with * are classified on family level, i.e., the genus is unknown. Numbers marked with ** are significantly different by Wilcoxons signed-rank test ($p < 0.05$).

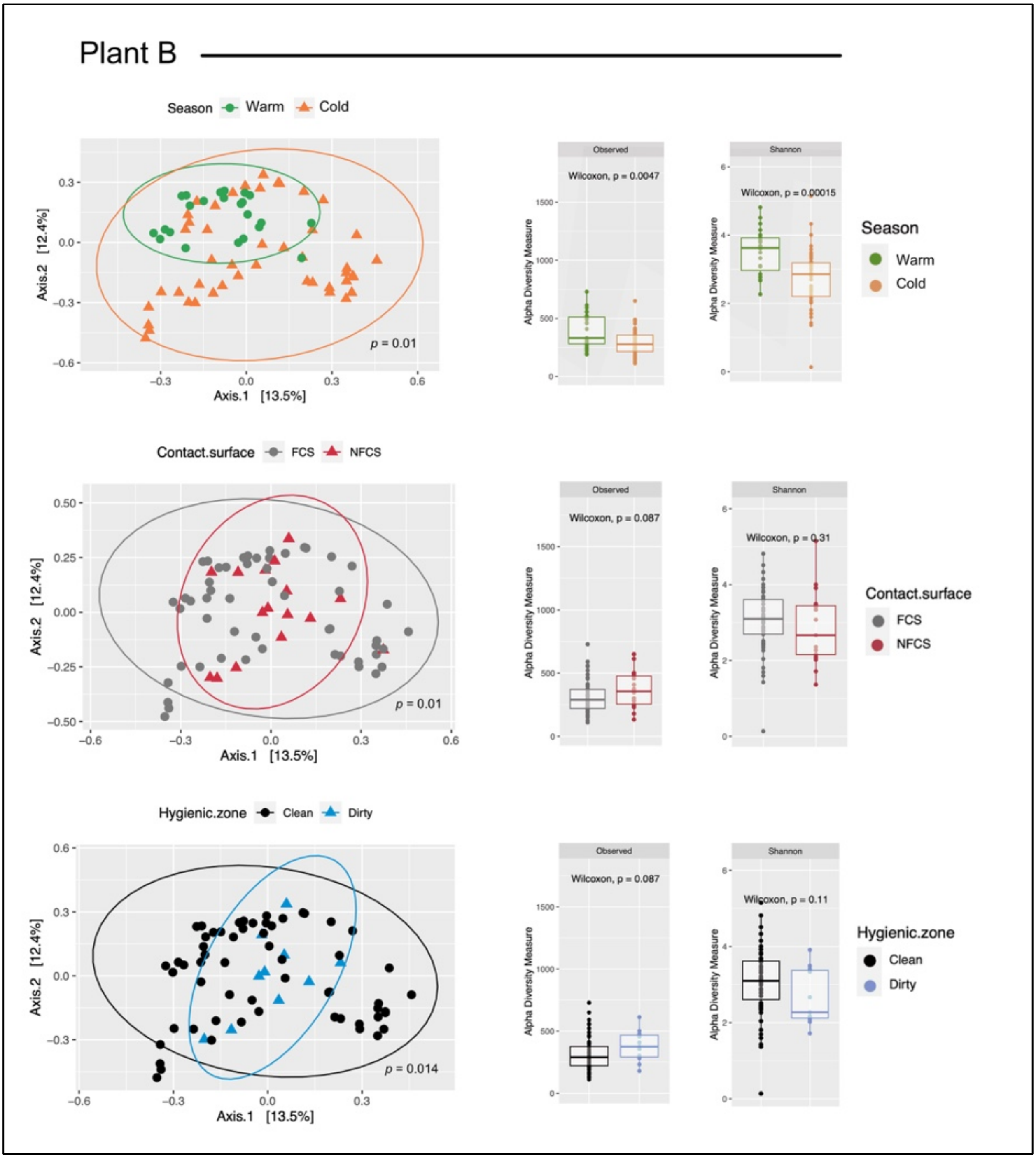

Fig. A6. Alpha- and beta-diversity in samples from Plant B: comparison of warm vs. cold season, FCS vs NFCS and clean vs. dirty hygienic zone. Alpha diversity is given by the Observed richness and the Shannon diversity index. Significant differences were calculated by Wilcoxons signed-rank test. While beta-diversity is shown by Principal Coordinates Analysis (PCoA) plots with Bray-Curtis dissimilarities and significant differences were analyzed using PERMANOVA (Adonis).

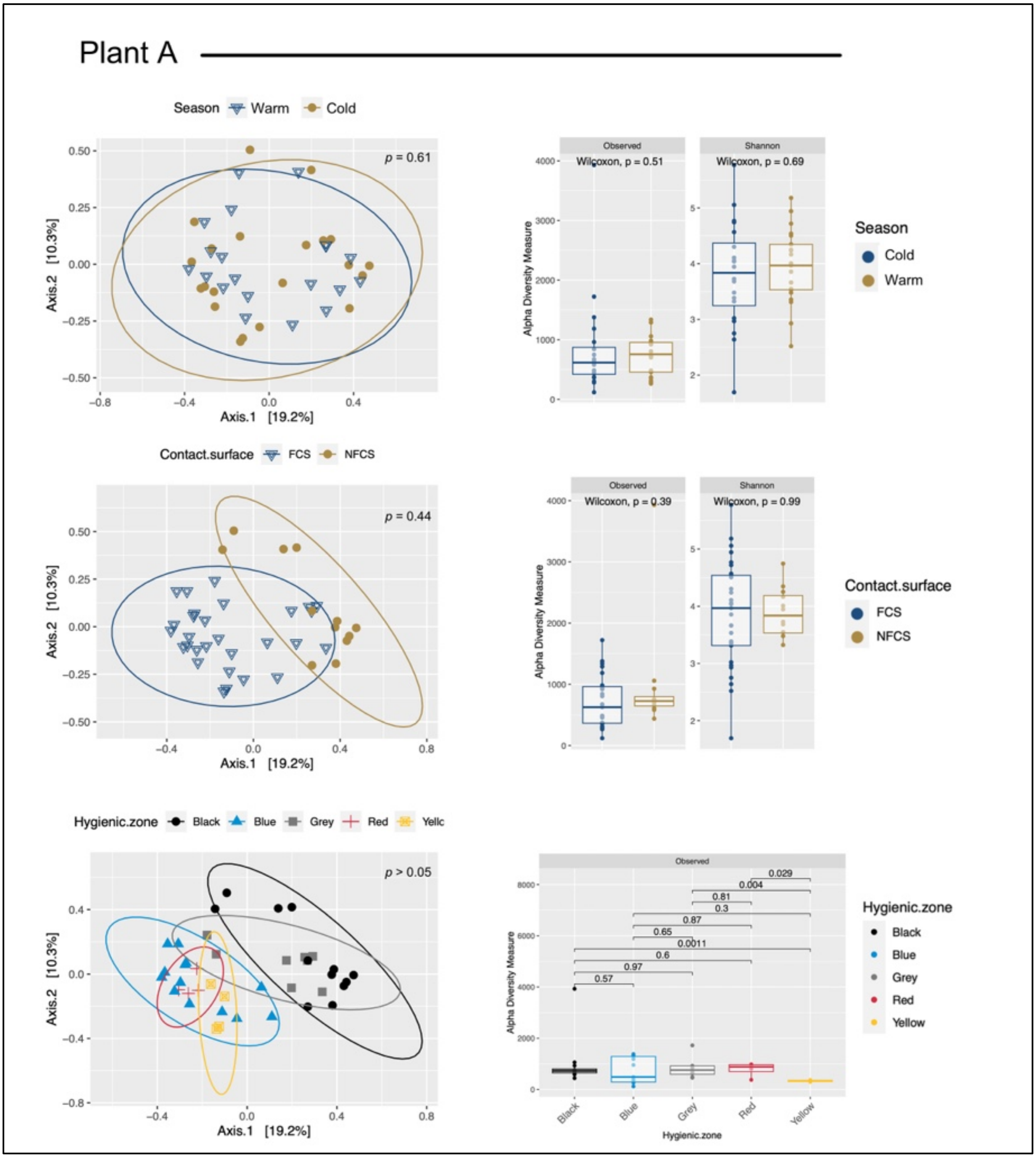

Fig. A7. Alpha- and beta-diversity in samples from Plant A: comparison of warm vs. cold season, FCS vs NFCS and clean vs. dirty hygienic zone. Alpha diversity is given by the Observed richness and the Shannon diversity index. Significant differences were calculated by Wilcoxons signed-rank test. While beta-diversity is shown by Principal Coordinates Analysis (PCoA) plots with Bray-Curtis dissimilarities and significant differences were analyzed using PERMANOVA (Adonis).

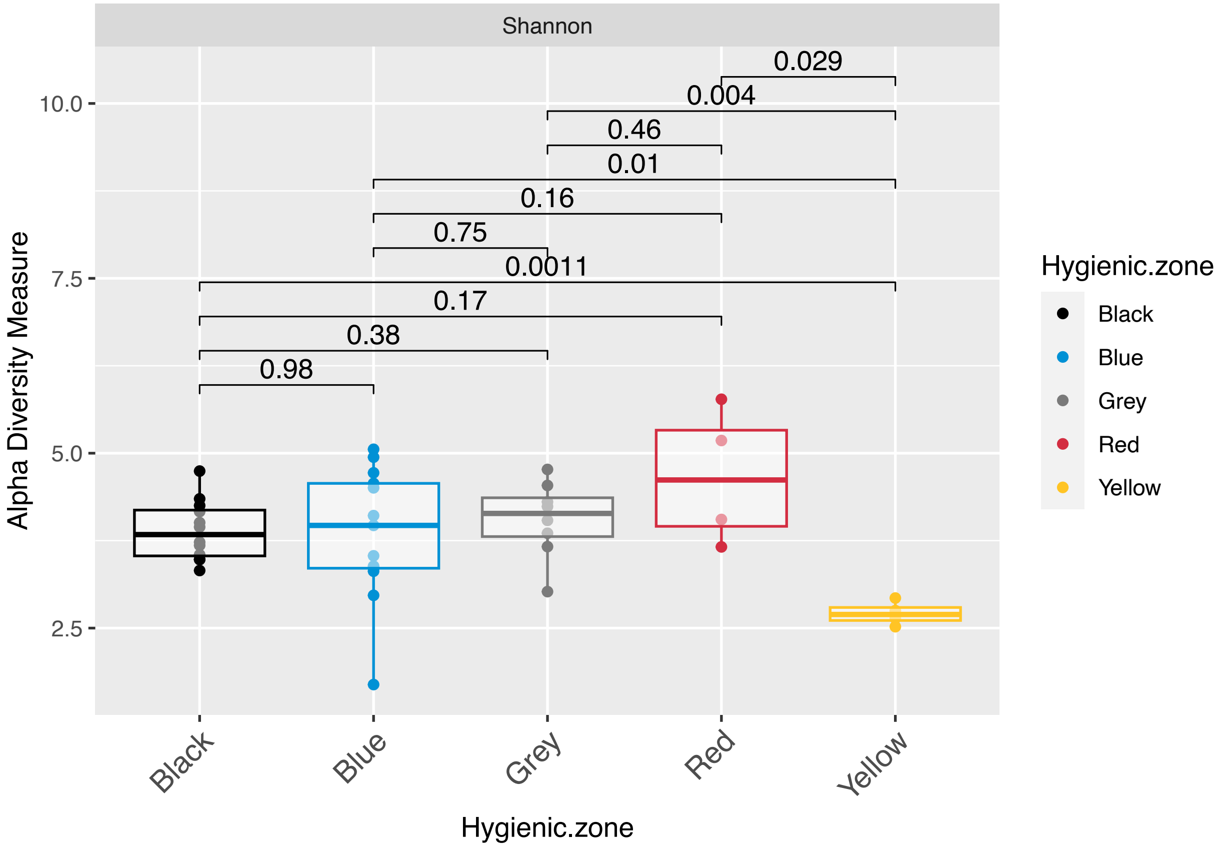

Fig. A8. Continuation of Fig A6: Shannon diversity in the black, blue, grey, red and yellow hygienic zone from Plant A. Significant differences were calculated by Wilcoxons signed-rank test.

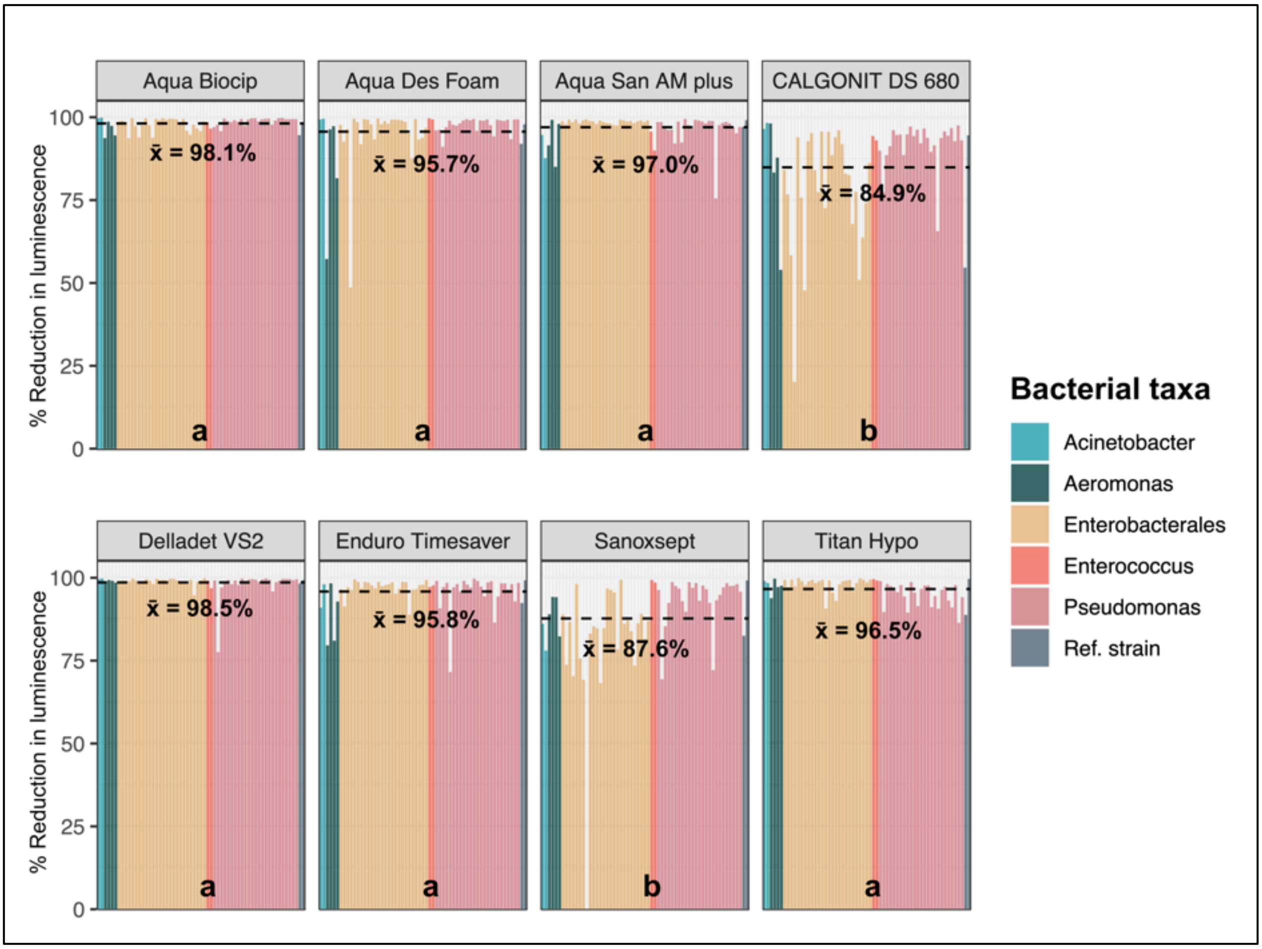

Fig. A9. Bar plots showing average % reduction of luminescence (RLU) in 62 biofilms after 20 min exposure to the highest recommended concentration of eight disinfectants. Each bar represents mean values of biofilm triplicates with color showing the taxonomic classification of the biofilm including *Acinetobacter* (n = 2), *Aeromonas* (n = 4), Enterobacterales (n = 27), *Enterococcus* (n = 2), *Pseudomonas* (n = 25) and references strains (n = 2). Different letters (a, b) are showing significant differences ($p = 0.05$) between the mean reduction % in luminesces of each disinfectant (calculated by Tukey PostHoc test).

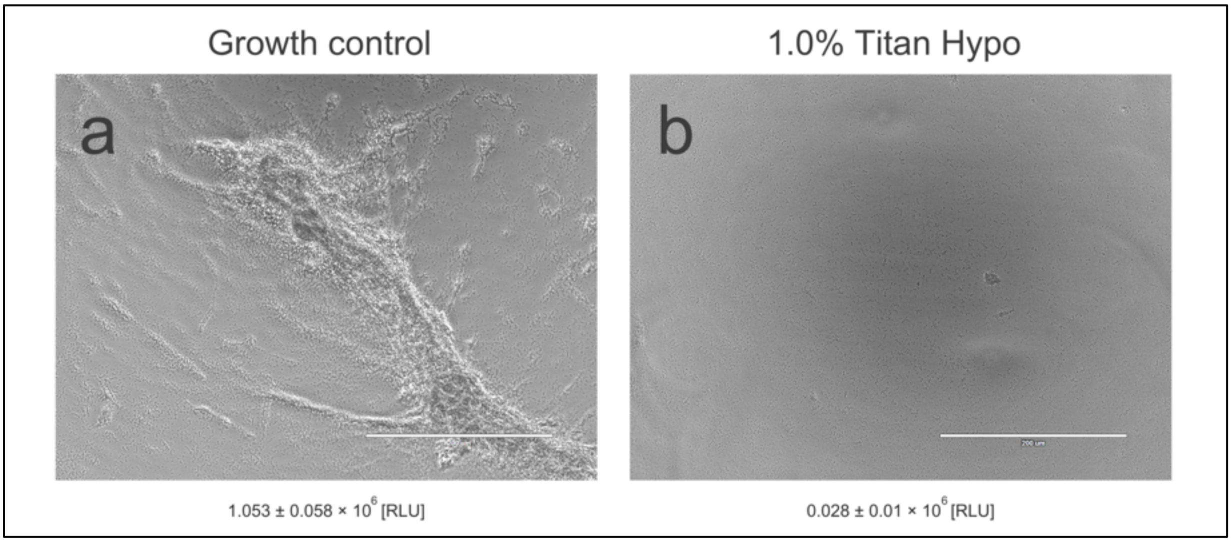

Fig. A10. Sectional micrograph of a biofilm growth control (a) by *Pseudomonas lundensis* (CNK7A-1-1*)* on the bottom of a 96 well plate after 24 hrs. A parallel biofilm exposed to 1.0% Titan Hypo for 20 min is shown in the sectional micrograph b. Taken by inverted phase contrast microscopy (EVOS® FL Auto Imaging system; Life Technologies™, Grand Island, USA). Image credit: Thorben Reiche.

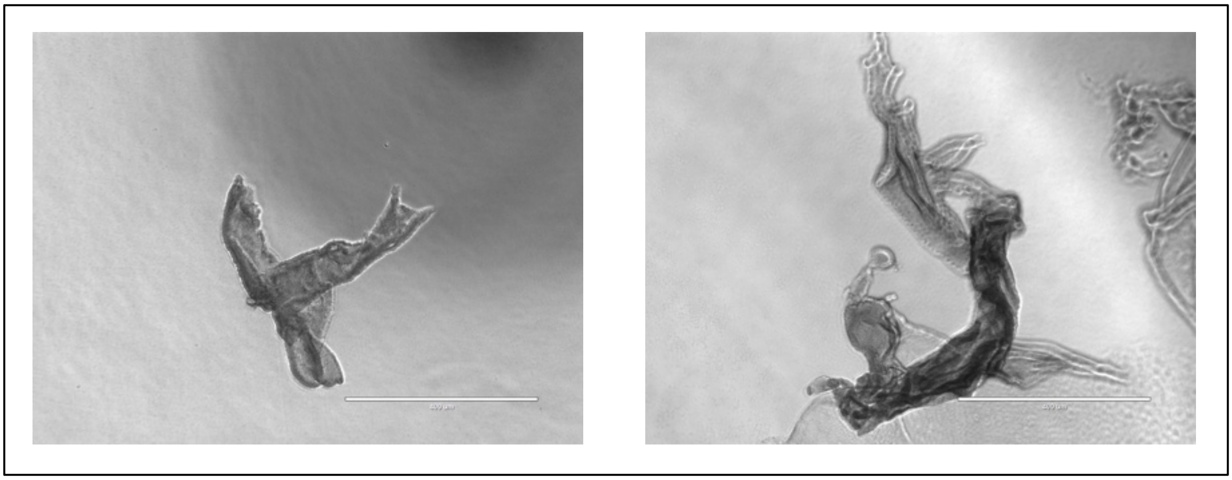

Fig. A11. Sectional micrograph of biofilm fragments observed in wells of *Pseudomonas aeruginosa* Ph5A-2-1 after 24 hrs. Taken by inverted phase contrast microscopy (EVOS® FL Auto Imaging system; Life Technologies™, Grand Island, USA). Image credit: Thorben Reiche.

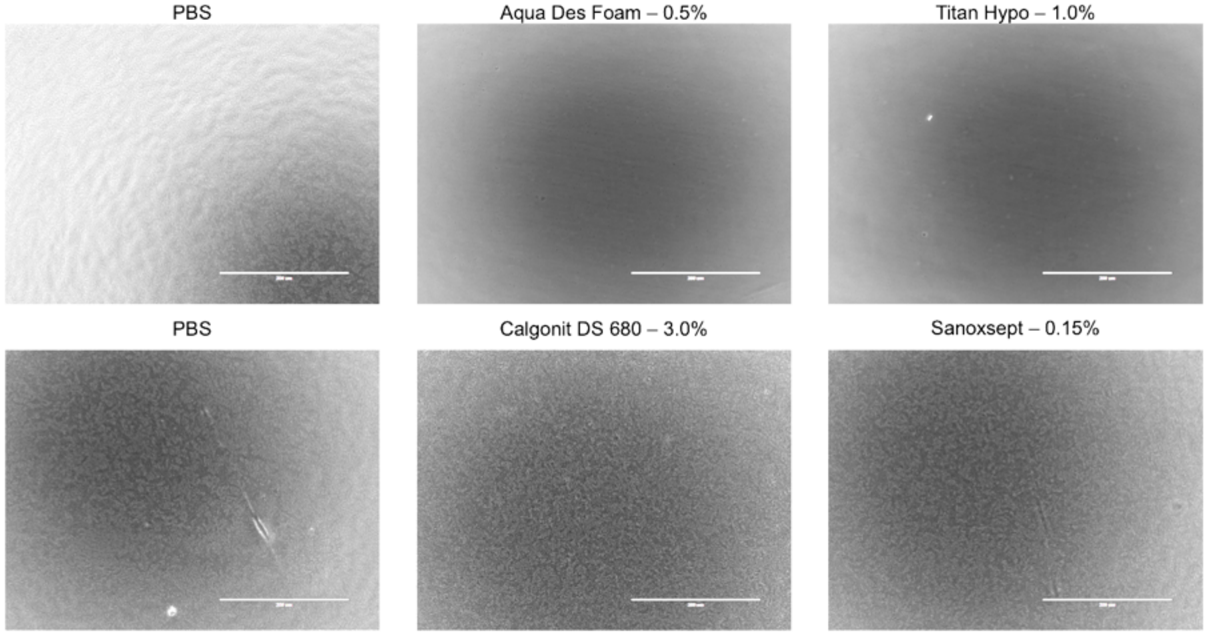

Fig. A12. Sectional micrographs of biofilms formed by *Hafnia paralvei* on the bottom of a 96 well plate after 24 hrs. Biofilm growth controls were exposed to PBS (phosphate buffered saline), while parallels were exposed to disinfectants Aqua Des Foam (0.5%), Titan Hypo (1.0%), Calgonit DS 680 (3.0%) and Sanoxsept (0.15%). Taken by inverted phase contrast microscopy (EVOS® FL Auto Imaging system; Life Technologies™, Grand Island, USA). Image credit: Thorben Reiche

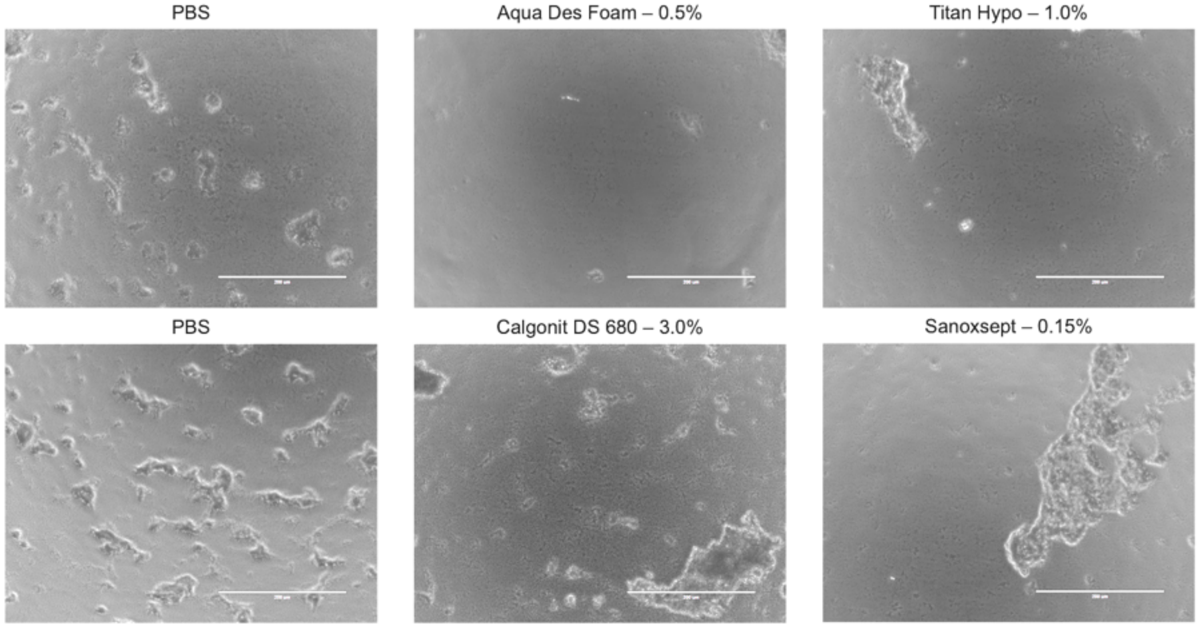

Fig. A13. Sectional micrographs of biofilms formed by *Pseudomonas fluorescens* on the bottom of a 96 well plate after 24 hrs. Biofilm growth controls were exposed to PBS (phosphate buffered saline), while parallels were exposed to disinfectants Aqua Des Foam (0.5%), Titan Hypo (1.0%), Calgonit DS 680 (3.0%) and Sanoxsept (0.15%). Taken by inverted phase contrast microscopy (EVOS® FL Auto Imaging system; Life Technologies™, Grand Island, USA). Image credit: Thorben Reiche

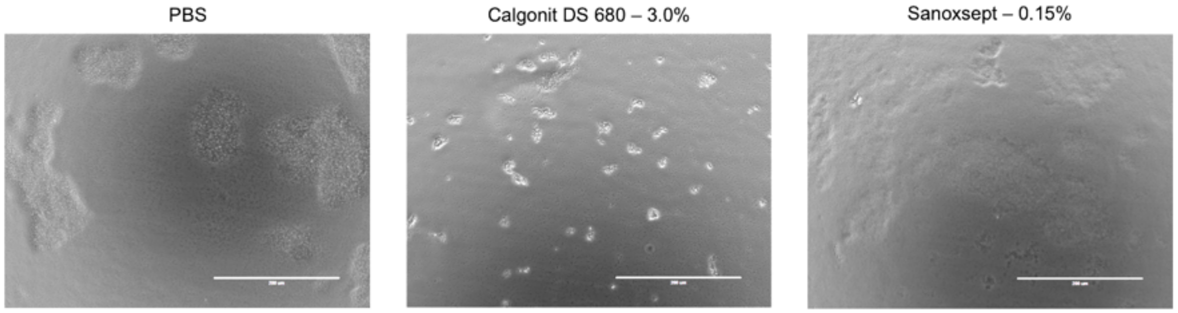

Fig. A14. Sectional micrographs of biofilms formed by *Pseudomonas otitidis* on the bottom of a 96 well plate after 24 hrs. Biofilm growth controls were exposed to PBS (phosphate buffered saline), while parallels were exposed to disinfectants Calgonit DS 680 (3.0%) and Sanoxsept (0.15%). Taken by inverted phase contrast microscopy (EVOS® FL Auto Imaging system; Life Technologies™, Grand Island, USA). Image credit: Thorben Reiche
